# Longitudinal study of care needs and behavioural changes in people living with dementia using in-home assessment data

**DOI:** 10.1038/s43856-024-00724-3

**Published:** 2025-01-10

**Authors:** Chloe Walsh, Alexander Capstick, Nan Fletcher-Lloyd, Jessica True, CR&T Group, David Sharp, Danielle Wilson, Sarah Daniels, David Wingfield, Matthew Harrison, Shlomi Haar, Mara Golemme, David Sharp, Martina Del Giovanne, Paresh Malhotra, Neil Graham, Emma Jane Mallas, Naomi Hassim, Greg Scott, Magdalena Kolanko, Alina-Irina Serban, Helen Lai, Eyal Soreq, Lucia M. Li, Tong Wu, Thomas Parker, Timothy Constandinou, Alan Bannon, Danilo Mandic, Charalambos, Adrien Rapeaux, Ghena Hammour, Ian Williams, Byran Hsieh, Maowen Yin, Niro Yogendran, Ravi Vaidyanathan, Ting Su, Maria Lima, Thomas Martineau, Mayue Shi, Melanie Jouaiti, Tianbo Xu, Maitreyee Wairagkar, Bo Xiao, Carlot Sebastian Castillo, Alehandro Valdunciel, Panipat Wattansiri, Reineira Seeamber, Annika Guez, Zehao Liu, Saksham Dhawan, Payam Barnaghi, Nan Fletcher-Lloyd, Amer Marzuki, Hamed Haddadi, Francesca Palermo, Mark Woodbridge, Anna Joffe, Yuchen Zhao, Samaneh Kouchaki, Alexander Capstick, Yu Chen, Tianyu Cui, Chloe Walsh, Paul Freemont, Loren Cameron, Thomas Adam, Michael Crone, Raphaella Jackson, Kristen Jensen, Martin Tran, Derk Jan Dijk, Anne Skeldon, Vikki Revell, Kevin Wells, Giuseppe Atzori, Ullrich Bartsch, Lucina Grainge, Ciro Della Monica, Hana Hassanin, Kiran GR Kumar, James Woolley, Damion Lambert, Iris Wood-Campar, Sara Mohammadi Mahvash, Janetta Rexha, Thalia Rodrigues Garcia, Shlomi Haar, Niro Yogendran, Subai Abulikemu, Julian Jeyasingh Jacob, Cosima Graef, Nathan Steadman, Akena Kutuzova, Federico Nardi, Assaf Touboul, Matthew Harrison, Lenny Naar, Sophie Horrocks, Brian Quan, David Wingfield, Naomi Hassim, Chloe Walsh, Ramin Nilforooshan, Jessica True, Olga Balazikova, Emily Beal, Nicole Whitethread, Matthew Purnell, Vaiva Zarombaite, Lucy Copps, Olivia Knight, Gaganpreet Bangar, Sumit Dey, Chelsea Mukonda, Jessia Hine, Luke Mallon, Claire Norman, Aanesha Patel, Ruby Lyall, Sanara Razall, Pippa Kirby, John Patterson, Mike Law, Andy Kenny, Ramin Nilforooshan, Payam Barnaghi

**Affiliations:** 1 Department of Brain Sciences, Imperial College London, London, UK; 2 Surrey and Borders Partnership NHS Foundation Trust, Leatherhead, UK; 3 UK Dementia Research Institute, Care Research and Technology Centre, London, UK

**Keywords:** Alzheimer's disease, Neurodegenerative diseases

## Abstract

**Background:**

People living with dementia often experience changes in independence and daily living, affecting their well-being and quality of life. Behavioural changes correlate with cognitive decline, functional impairment, caregiver distress, and care availability.

**Methods:**

We use data from a 3-year prospective observational study of 141 people with dementia at home, using the Bristol Activities of Daily Living Scale, Neuropsychiatric Inventory and cognitive assessments, alongside self-reported and healthcare-related data.

**Results:**

Here we show, psychiatric behavioural symptoms and difficulties in activities of daily living, fluctuate alongside cognitive decline. 677 activities of daily living and 632 psychiatric behaviour questionnaires are available at intervals of 3 months. Clustering shows three severity-based groups. Mild cognitive decline associates with higher caregiver anxiety, while the most severe group interacts more with community services, but less with hospitals.

**Conclusions:**

We characterise behavioural symptoms and difficulties in activities of daily living in dementia, offering clinically relevant insights not commonly considered in current practice. We provide a holistic overview of participants’ health during their progression of dementia.

## Plain language summary

People with dementia experience changes in behaviour, independence and memory that impacts their own lives and the lives of the people who care for them. We study how living with dementia affects daily life and well-being after dementia diagnosis. In total, 141 people with dementia participated in the study. Study participants and their carers answered questions about daily activities, mental health, and cognitive abilities every 3 months. We found that behavioural changes and challenges in daily tasks for people with dementia are affected as memory and thinking skills decline. Carers of people with mild symptoms of dementia reported higher levels of stress, while people with more severe symptoms relied more on community services with a lower number of hospital visits. These findings offer insights to improve dementia care and support services.

## Abstract (web summary)

Walsh et al. examine how dementia impacts daily life and well-being over three years by tracking changes in behaviour, memory, and daily activities, for people with dementia and their carers. They find that as cognitive decline progresses, daily challenges increase, affecting carer stress and the use of community services.

## Introduction

Dementia is characterised by an acquired, progressive loss of executive function and cognitive abilities that are severe enough to interfere with everyday life^1^. Dementia presents a pressing global health challenge, exerting profound impact on affected individuals, their families or relatives and the wider healthcare systems^2^. In particular, behavioural and psychological symptoms have been recognised as significant aspects that should be assessed and therefore, managed as part of dementia care^3^.

A higher prevalence of neuropsychiatric symptoms has been associated with progression of cognitive decline^4^ and more severe psychiatric symptoms such as delusions and hallucinations have been associated with particular dementia diagnoses^5,6^. These behaviours exert widespread consequences on People Living with Dementia (PLWD), act as primary stressors for family and carers^7–9^ and are profoundly resource-intensive to the wider healthcare services^3^, throughout diagnosis. Severe behavioural and psychological symptoms have been associated with greater cognitive severity^10,11^ and with exponential increases to cost of, interventions and hospitalisations^12^. These symptoms can include: hallucinations, delusions, apathy, depression and anxiety. However, other external stressors can contribute to high individual variability of behavioural and psychological symptoms^13^. The Neuropsychiatric Inventory (NPI) is a research tool used to measure behavioural and psychological symptoms of PLWD^14–16^.

Difficulties in activities of daily living can contribute to loss of independence and behavioural changes in PLWD, leading to individuals becoming heavily reliant on carers and/or family members^17–19^. In turn, decline in executive functioning of PLWD can lead to loss of ability to perform basic/instrumental activities of daily living^20^. These activities can include: dressing, washing, eating, mobility and managing finances. Impairment in performance of these activities can be quantified using the research tool, Bristol Activities of Daily Living Scale (BADL)^21^.

The Lancet Commission on Dementia in 2020 identified the need for a consideration of dementia care as a whole, with regards to multiple aspects including cognitive, psychological, environmental and social needs^22^. This work also identified possible factors such as comorbidities that may contribute to how dementia care can be managed. Understanding of different factors that affect dementia care, can pave the way to personalised interventions with a greater benefit for individuals and their families^23^. The Lancet Commission on Dementia by Livingston et al.^24^ emphasises the need for using innovative approaches to understand dementia progression and the role of personalised care. Specifically, the commission calls for increased use of real-world data and longitudinal studies to improve our understanding of prognostic outcomes of dementia. This study focuses on this unmet clinical and care need and provides an analysis of the progression of PLWD. Combining in-home assessments with healthcare interactions, we offer insights into how real-world, everyday functional decline influences quality of life and prognosis for PLWD^24^.

Many studies that measure changes in behaviour of PLWD are either set in long-term care facilities^16^, hospital settings^25^ or use interventions such as medications^26–28^. Typically, only singular aspects are assessed during longitudinal follow-up studies such as cognition over a long period of time^29^. Little evidence exists to solely evaluate the changes in either cognition, behaviour and/or function alongside other clinical information. Some examples of longitudinal cohorts of PLWD such as the 4C study, have taken a similar approach to our work in terms of regular assessments and a wider view of participants’ health status however, these were only conducted every 12 months, nonetheless in a larger cohort^30^. Highlighted here, was the importance of understanding trajectories in dementia, longitudinally, to benefit patient care^30^. Inability to identify changes or decline in function at earlier stages leaves clinicians at a loss in how to manage them. If regularly monitored and identified, the progressive interplay between cognitive decline, behaviour and daily functioning can be mapped to allow for more timely intervention, reducing cost of care to both families and hospitals^31,32^.

Remote monitoring solutions and Internet of Things technologies have begun to shift dementia care to begin at home in the community of PLWD before reaching severe stages. Previous work from our group has shown how data from in-home technology and methods in machine learning can be used for pattern analysis^33–35^.

In this work, we specifically explore how activities of daily living, behavioural and psychological symptoms of participants in our cohort relates to cognition and how this relationship can inform holistic, personalised interventions. We integrate data gathered from assessments and wider healthcare records and apply machine learning techniques to identify three severity-based groups in the cohort. This study offers a novel approach to using in-home assessments to identify subgroups of PLWD according to their care needs and presents a pivotal step towards a more nuanced understanding of dementia care, stressing the need for a broader and comprehensive, individualised approach in dementia care. Most recent studies in this domain, focus on prognosis of clinical pathology and clinical symptoms of dementia^36,37^. However, while clinical findings are important, the most impactful interventions to support PLWD depends on a more accurate assessment of their care requirements.

In this study, we use clinically validated assessments to identify care needs of patient subgroups, using routinely collected in-home assessment data. This offers a highly scaleable and generalisable approach, that can also be applicable to low-resource healthcare settings. Using machine learning models and this longitudinal data we have identified groups of PLWD with distinct trajectories of decline and care requirements. This will allow for a more clinically applicable mapping of individuals’ trajectory throughout their dementia diagnosis, using clinically available routine assessments.

## Methods

### Study design and data collection

Data used in this study is from the Minder Health Management Study (IRAS: 257561), spanning over three years (24/07/2020 to 01/09/2023). Inclusion criteria for participation were frail adults and/or people living with neurological conditions/neurodegenerative diseases, including dementia, stroke and traumatic brain injury. Exclusion criteria included people with severe depression, severe psychosis, agitation and anxiety at screening and baseline. People with severe sensory impairment, active suicidal ideas and those receiving treatment for terminal illness at baseline were also excluded. For the purpose of this work, we only include participants with a diagnosis of dementia (*n* = 141).

Participants were recruited from primary care, adult social care services and memory clinics across Surrey and Borders Partnership National Health Service (NHS) Foundation Trust and Hammersmith & Fulham Health and Care Partnership, North West London. This cohort included participants who had a confirmed diagnosis of dementia, a total of 141. Participants completed a dementia proforma at baseline, that consisted of information about demographics, including age and sex. Participants and proxy-raters completed multiple, regular assessment scales however, only five relevant assessment scales were selected for analysis. Fortnightly telephone calls were conducted to collect data about self-reported changes in behaviour and any interactions with healthcare services. We also used electronic healthcare record data to verify diagnoses and incidences of comorbidities for each participant.

### Ethical statement

The study was approved by the Health Research Authority’s London-Surrey Borders Research Ethics Committee (19/LO/0102). All participants provided written informed consent for participation and for their data to be included in publications. This included participants, study partners (referred to here as, proxy-raters) or study support key workers (proxy-raters). At baseline, participants and proxy-raters signed a consent form, if any lacked capacity, a personal or professional consultee was sought. Consultees are asked to provide an opinion on the views and feelings they believe the participant would have towards participation in the study. This is recorded on a consultee declaration form, as approved by the London-Surrey Borders Research Ethics Committee^38^. We followed the Health Research Authority (HRA) UK guidelines: ‘Personal consultee, i.e. a person who cares for the adult lacking capacity or is interested in that person’s welfare but is not doing so for remuneration or acting in a professional capacity’.

In the first instance, a personal consultee was sought and in the majority of cases this person also acted as the proxy-rater. However, there were cases where there was no personal consultee available therefore, a professional consultee (with no links to the study) was sought; this was a person who had power of attorney. The professional consultee often acted as the ‘Study Support Key Worker’ (proxy-rater). In the cases where a consultee was sought, personal or professional, a separate consultee declaration form was used, in addition to the consent form for proxy-raters.

### Pre-processing of data

All assessment data were pre-processed. Any diagnoses that had been re-evaluated by the research team, (made of neurologists, old age psychiatrist and neuroradiologists) following an Magnetic Resonance Imaging (MRI) scan and cognitive testing, replaced existing diagnoses from electronic healthcare records (*n* = 20).

Missing assessment time points were removed from the data. Pre-processing of Neuropsychiatric Inventory data included calculating the symptom ‘frequency’ multiplied by symptom ‘severity’ to determine a total raw score for each symptom; as part of the scale, frequency and severity of each symptom are summed to produce the Neuropsychiatric Inventory total score. Neuropsychiatric Inventory distress was recorded separately. Missing assessments with values less than 50% were imputed with 0 to indicate no symptom was present for both Neuropsychiatric Inventory and Bristol Activities of Daily Living Scale. Any participants with more than 50% of the assessment missing were not included. No further imputation of assessment data was used in this analysis.

### Assessments

Regular assessments were conducted by research assistants and included two standardised, validated cognitive assessments, the Alzheimer’s Disease Assessment Scale Cognitive Sub-scale (ADAS-Cog) (14-item) every 6 months; and the Standardised Mini-Mental State Examination (SMMSE) every 12 months. Scaling of the cognitive assessments differs such that, the more severe the cognitive impairment, the higher the ADAS-Cog score; whereas, the lower the score for SMMSE, the more severely cognitively impaired the individual.

In addition, we used the previously validated Bristol Activities of Daily Living Scale as an assessment of activities of daily living and the Neuropsychiatric Inventory as a measure of psychiatric behavioural symptoms of PLWD. From herein, we will refer to the Bristol Activities of Daily Living Scale (BADL) as the activities of daily living assessment and the Neuropsychiatric Inventory (NPI) as the psychiatric behaviour assessment. The psychiatric behaviour assessment (Frequency*Severity) accounts for the neuropsychiatric symptoms that each participant experiences whereas, the psychiatric behaviour assessment distress score measures the proxy-rater distress according to how severe and frequent the symptoms of the participant are. Both the activities of daily living and psychiatric behaviour assessments were about the participant, however, were completed by the proxy raters.

Study partners were defined as someone who had known the participant for at least 6 months and had a good existing relationship with the participant. Different study partners included spouses, children, neighbours, friends and other relatives. No professional carers were assigned as study partners instead, these were asked to join the study as Study Support Key Workers.

Any participants without a study partner, were not included in this analysis however, they still remained in the main study. For assessments, the study partners acted as proxy-raters. The Hospital Anxiety and Depression Scale was the only assessment analysed here, that assessed proxy-raters. From herein, we will refer to the Hospital Anxiety and Depression Scale as the proxy-rater well-being assessment. The activities of daily living, psychiatric behaviour and proxy-rater well-being assessments were conducted every 3 months to gain greater granularity of changes in participants during the study.

### Interactions with healthcare services

Data was regularly collected from electronic healthcare records for each participant where possible, regarding their comorbidities and medications. As amendments were submitted throughout the study with changes to patient information sheets and consent forms, only 93 participants consented to retrospective access to health care records. Interactions with healthcare services and changes in behaviour were self-reported during the regular check-in calls with participants. We categorised comorbidities, according to categories adapted from the population-based study from Kuan et al.^39^. These interactions and self-reported behaviours were encoded so that ‘1’ represented an encounter or presentation of behaviour and ‘0’ was a lack thereof.

### Statistics and reproducibility

According to the data distributions, appropriate descriptive statistics are reported. Correlation analysis of assessments was conducted using Spearman’s Rank Correlation. For further analysis, to account for differences in scales and how they quantify presentation, or lack thereof, a behaviour, we normalised the responses from the activities of daily living and psychiatric behaviour assessments using binary outcomes. A score of ≥1 indicated presence of each neuropsychiatric symptom, within the last month (psychiatric behaviour assessment) or difficulty with that component/activity of daily living, in the last 2 weeks (activities of daily living assessment).

To compare clusters, we employed a combination of non-parametric statistical methods to determine statistically significant differences. Firstly, a Kruskal–Wallis test was used to compare the overall differences in scores for each feature, to examine group-wise differences. A significance threshold of 0.05 was applied. We then used Dunn’s post-hoc testing to identify the pairwise differences between each cluster, per feature. Similarly, a significance threshold of 0.05 was applied here. Bonferroni multiple comparisons correction was used for corresponding *p* values.

### Machine learning

A clustering method was used to determine whether we could evaluate the progression of the cohort based on their baseline scores in difficulties of activities of daily living and neuropsychiatric symptoms. Therefore, baseline activities of daily living and psychiatric behaviour assessment scores were chosen as features for the clustering.

All analysis used in this work was done using Python (V3.9.13) and libraries used included Scikit-Learn version: 1.1.3, Pandas version: 1.5.1, NumPy version: 1.23.4 and visualisation was done using Seaborn version: 0.11.2. In total, 87 initial activities of daily living and psychiatric behaviour assessments were used in the clustering. Overall, 35 different features were used in the clustering model made up of all activities of daily living and psychiatric behaviour assessment questions/components. Features of the models were scaled using a robust scalar and power transformer. Principal Component Analysis was then applied to reduce the features from 35 to 15, a 43% reduction. We used a selection of unsupervised clustering techniques including K-Means (distanced-based), Gaussian Mixture Modelling (distribution-based), Bayesian Gaussian Mixture Modelling (probabilistic-based) and Hierarchical Agglomerative Clustering. For each method, we used between two and seven cluster groups, tuned to specific hyper-parameters. To evaluate the clustering performance, Calinski-Harabasz Index^40^ was used, this evaluates how well-defined the clusters are and a higher index indicates groups are distinct and well-separated. Three other metrics were also included; Davies-Bouldin Score, assesses the average similarity between each cluster and its most similar cluster^41^, the Dunn Index which measures the compactness of clusters and separation between clusters therefore, the lower the index the more well separated^42^ and the Silhouette Score that measures how similar an object is to its own cluster compared to other clusters^43^.

### Reporting summary

Further information on research design is available in the Nature Portfolio Reporting Summary linked to this article.

## Results

### Exploring daily living activities and psychiatric behaviour

Data from 141 PLWD were available. Characteristics of our cohort are displayed in Table 1. The density and distribution of each scale are displayed in Supplementary Notes, Supplementary Fig. 1. Baseline scores were correlated against one another (Supplementary Fig. 2). A total of 677 activities of daily living assessments, 632 psychiatric behaviour assessments and 646 proxy-rater well-being assessments were available in the cohort. 109 participants had completed 296 Alzheimer’s Disease Assessment Scale-Cognitive Subscale (ADAS-Cog) scales and 118 participants had completed 194 Standardised Mini-Mental State Examination (SMMSE) scales. 87 baseline activities of daily living, behaviour and proxy-rater well-being assessments were completed. We correlated each assessment score with the cognitive scales, ADAS-Cog and SMMSE, conducted at the same time-point (Tables 2, 3). When assessed at the same assessment time-point, all scales except the proxy-rater well-being assessment were positively correlated with the ADAS-Cog Table 2) and inversely correlated with the SMMSE (Table 3).

**Table 1:** Summary of cohort demographics and diagnoses

Dementia diagnosis	Sex		Age group					House occupancy		Count (*n* = 141)	% of Total cohort
	%Male	%Female	%50–59	%60–69	%70–79	%80–89	%90+	%Lives alone	%Lives with proxy-rater		
Alzheimer’s Disease	48.39	51.61	…	6.45	27.96	47.31	18.28	41.94	53.76	93.00	65.96
Mild cognitive impairment	36.36	63.64	…	…	…	81.82	18.18	45.45	54.55	11.00	7.80
Other	66.67	33.33	5.56	11.11	55.56	22.22	5.56	44.44	52.78	37.00	26.24

Summary statistics of the current cohort based on diagnosis for sex, age group and occupancy in the home. To preserve privacy of participants we categorised all other dementia diagnoses apart from Alzheimer’s Disease and Mild Cognitive Impairment as ‘Other’.

**Table 2:** Correlation of Alzheimer’s disease assessment scale - cognitive subscale with in-home assessments

	ADAS-Cog		NPI (Frequency*Severity)		NPI (proxy-rater distress)		BADL		Proxy-rater HADS	
	*r*	*p* Value	*r*	*p* Value	*r*	*p* Value	*r*	*p* Value	*r*	*p* Value
ADAS-Cog	1.00	0.00	0.33	4.43e–08	0.23	1.25e–04	0.66	9.87e–35	0.19	1.67e–03
NPI (Frequency*Severity)	0.33	4.43e–08	1.00	0.00	0.84	5.29e–72	0.44	7.31e–14	0.44	3.88e–14
NPI (Proxy-rater Distress)	0.23	1.25e–04	0.84	5.29e–72	1.00	0.00	0.40	2.71e–11	0.50	9.04e–18
BADL	0.66	9.87e–35	0.44	7.31e–14	0.40	2.71e–11	1.00	0.00	0.35	6.85e–09
Proxy-rater HADS	0.19	1.67e–03	0.44	3.88e–14	0.49	9.04e–18	0.35	6.85e–09	1.00	0.00

Results of Spearman’s rank correlation coefficient of corresponding assessments with 6-monthly cognitive assessment ADAS-Cog, conducted at the same time-point (*n* = 262). NPI refers to the psychiatric behaviour assessment, NPI distress is proxy-rater distress associated with participants’ psychiatric behaviour, BADL refers to the activities of daily living assessment and HADS refers to well-being of proxy-rater. (*r* = Spearman’s correlation coefficient).

*ADAS-Cog* Alzheimer’s Disease Assessment Scale Cognitive Subscale, *NPI* Neuropsychiatric Inventory, *BADL* Bristol Activities of Daily Living, *HADS* Hospital Anxiety and Depression Scale.

**Table 3:** Correlation of standardised mini-mental state examination with in-home assessments

	SMMSE		NPI (Frequency*Severity)		NPI (proxy-rater distress)		BADL		Proxy-rater HADS	
	*r*	*p* Value	*r*	*p* Value	*r*	*p* Value	*r*	*p* Value	*r*	*p* Value
SMMSE	1.00	0.00	−0.31	4.15e–05	−0.24	2.17e–03	−0.59	7.56e–17	−0.13	0.092
NPI (Frequency*Severity)	−0.31	4.15e–05	1.00	0.00	0.82	1.35e-42	0.42	2.02e–08	0.40	9.63e–08
NPI (Proxy-rater Distress)	−0.24	2.17e–03	0.82	1.35e–42	1.00	0.00	0.38	4.87e–07	0.51	1.69e–12
BADL	−0.59	7.56e–17	0.42	2.02e–08	0.38	4.87e–07	1.00	0.00	0.37	9.86e–07
Proxy-rater HADS	−0.13	0.092	0.40	9.63e–08	0.51	1.69e–12	0.37	9.86e–07	1.00	0.00

Results of Spearman’s rank correlation coefficient of corresponding assessments with annual cognitive assessment SMMSE, conducted at the same time-point (*n* = 167). NPI refers to the psychiatric behaviour assessment, NPI distress is proxy-rater distress associated with participants’ psychiatric behaviour, BADL refers to the activities of daily living assessment and HADS refers to well-being of proxy-rater. (*r* = Spearman’s correlation coefficient).

*SMMSE* Standardised Mini-Mental State Examination, *NPI* Neuropsychiatric Inventory, *BADL* Bristol Activities of Daily Living, *HADS* Hospital Anxiety and Depression Scale.

**Fig. 1:**
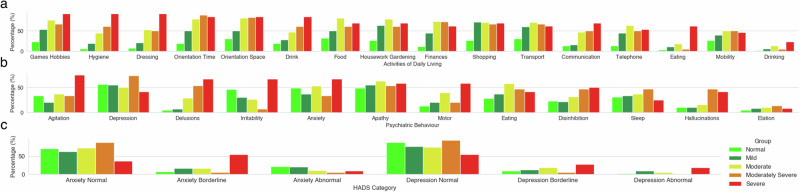
Prevalence of difficulties in activities of daily living and psychiatric behaviours of the cohort relative to cognitive scores and how they relate to proxy-rater well-being. **a** Activities of daily living assessment (BADL), (**b**) psychiatric behaviour assessment (NPI) and (**c**) proxy-rater well-being assessment (HADS) according to the participants' cognitive score in the SMMSE (*n* = 119). **a** In the activities of daily living assessment, a score of ≥1 indicated difficulty with that component/activity of daily living, in the last 2 weeks. **b** For the psychiatric behaviour assessment, a score of ≥1 indicated presence of each neuropsychiatric symptom, within the last month. Frequency and severity of the symptom is not illustrated here. **c** From the proxy-rater well-being assessment, we show the percentage of either normal, borderline or abnormal anxiety and depression of proxy-raters, that correspond to participants with either normal, mild, moderate, moderately severe or severe SMMSE scores. NPI refers to the psychiatric behaviour assessment, BADL refers to the activities of daily living assessment and HADS refers to well-being of proxy-rater. SMMSE Standardised Mini-Mental State Examination, NPI Neuropsychiatric Inventory, BADL Bristol Activities of Daily Living, HADS Hospital Anxiety and Depression Scale.

Participants’ activities of daily living and neuropsychiatric behaviour varied, relative to their corresponding cognitive ability (Fig. 1). SMMSE scores, taken within 3 months of the activities of daily living, behaviour and proxy-rater well-being assessments were included (*n* = 87). Severity of the SMMSE scoring is categorised based on NICE guidelines recommendation^44^. Figure 1 illustrates the sum or incidence of participants that scored difficulties in each question for (a) activities of daily living assessment, (b) psychiatric behaviour assessment and (c) proxy-rater well-being assessment, according to the severity of the corresponding SMMSE score. In this sub-group, of all SMMSE scales conducted, 11 were severe, 24 were moderately severe, 37 moderate, 61 mild and 41 normal.

The most prevalent difficulties reported in activities of daily living as measured by the activities of daily living assessment, were housework/gardening, shopping and preparing food (Fig. 1a). Difficulties in housework/gardening, preparing food and shopping were reported for at least six consecutive months for 65% of the cohort. Severe SMMSE participants most commonly reported difficulty with dressing selves, taking part in games/hobbies and maintaining hygiene. Mild SMMSE scores corresponded to a higher frequency of difficulty with shopping whereas, normal scoring participants most frequently reported difficulties with transport.

Apathy, depression and anxiety were the most common neuropsychiatric symptoms reported across the cohort (Fig. 1b). Changes in eating and sleep behaviours persisted for at least six consecutive months in over half of the cohort. Sleep behaviour changes were also reported in all severity groups at a similar frequency.

Figure 1c illustrates the percentage of proxy-raters, that scored either normal, borderline or abnormal in both anxiety and depression, corresponded to participants’ SMMSE score. Normal levels of depression were seen for at least 6 months in 55% of proxy-raters but only 48% reported normal levels of anxiety for at least 6 months. Interestingly, the levels of abnormal depression and anxiety do not follow a clear linear relationship as participants progress in severity. The more severe the SMMSE, the more frequently proxy-raters were experiencing abnormal levels of depression however, abnormal levels of anxiety decreased from mild to moderate and then increased again to moderately severe. Borderline levels of depression and anxiety for at least six consecutive months were 15% and 11%, respectively. On the other hand, abnormal levels of anxiety were higher than abnormal levels of depression, in proxy-raters for at least six consecutive months (15% and 5%, respectively).

### Clustering baseline activities of daily living and psychiatric behaviour scores

Machine learning techniques enable us to detect patterns otherwise lost in the complexity of real-world data. In this work, we utilised clustering techniques to identify how our cohort could be divided according to their baseline activities of daily living and neuropsychiatric symptoms, whilst unpacking the broader healthcare-related data that encompassed each individual’s journey in their dementia diagnosis.

We used a variety of methods to determine the most appropriate clustering method that was of greatest clinical relevance (see Supplementary Notes—Evaluation of Clustering, Supplementary Table Supplementary Table 2).

**Fig. 2:**
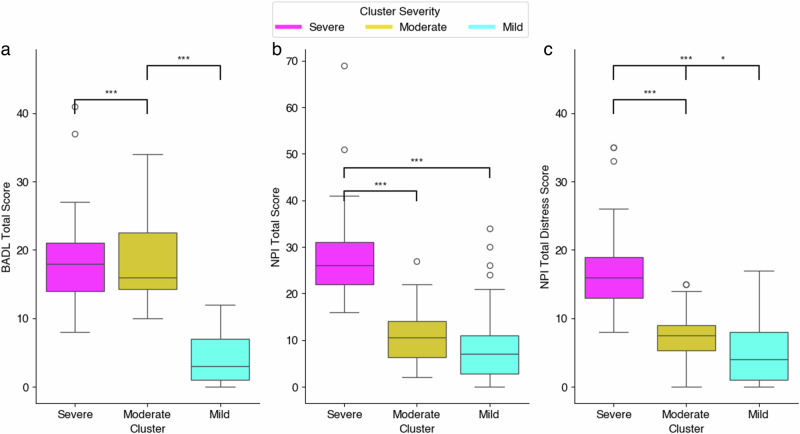
In-home assessment scores per cluster group at baseline for activities of daily living, psychiatric behaviours and proxy-rater well-being. Baseline total activities of daily living and psychiatric behaviour assessment scores per cluster from K-Means clustering model (Calinski-Harabasz Score: 18.52). Each cluster is described on the *x*-axis, Severe, Moderate and Mild. Model features included, but were not limited to, baseline (**a**) activities of daily living assessment (*n* = 87), (**b**) total behaviour (*n* = 87) and (**c**) total behaviour proxy-rater distress scores (*n* = 87). Kruskal–Wallis test was conducted to compare cluster groups and post-hoc Dunn’s test was conducted to investigate group differences. Significance threshold was set at *p* value < 0.05; exact *p* values can be found in Supplementary Table 4. (* <0.05; ** <0.01; *** <0.001). NPI refers to the psychiatric behaviour assessment, NPI distress is proxy-rater distress associated with participants' psychiatric behaviour and BADL refers to the activities of daily living assessment. NPI Neuropsychiatric Inventory, BADL Bristol Activities of Daily Living.

The mean scores and standard deviations for the features of each cluster are displayed in Supplementary Table Supplementary Table 3. A summary table of the comparison of the cluster pairings is illustrated in Supplementary Table Supplementary Table 4. The total scores for activities of daily living, psychiatric behaviour assessment and behaviour symptom distress are illustrated in Fig. 2. It is clear from this grouping that the clustering divided the group based on their overall performance in these scales nonetheless, significant differences were only consistent across all three clusters for the psychiatric behaviour assessment distress score of the proxy-raters (see Supplementary Table Supplementary Table 4). For the activities of daily living and psychiatric behaviour assessment total scores, cluster 0 had a significantly higher score in all scores, compared to cluster 1 (*p* < 0.001) but, not with cluster 2. Therefore, from the performance of the clustering it is clear that cluster 0 is distinctly the highest-scoring (most severe) group and cluster 1, the lowest-scoring (least severe) group. For simplicity, we have named each cluster according to this ranking, to better understand their context. We will refer to each cluster using the following descriptions: Cluster 0—Severe cluster, Cluster 1—Mild cluster, Cluster 2—Moderate cluster.

### Profiling clusters—assessment scores

**Fig. 3:**
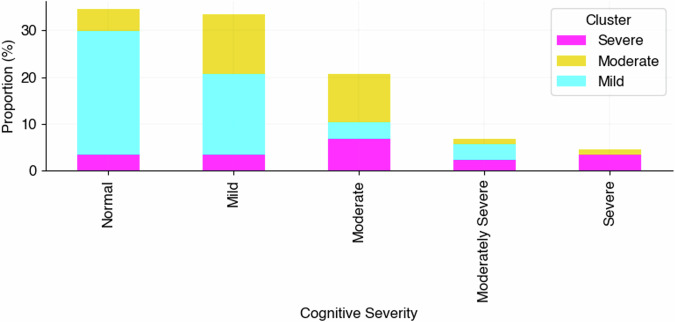
Proportion of standardised mini-mental state examination cognitive severity scores per cluster group at baseline. We illustrate the proportion of participants in each cluster that scored either normal, mild, moderate, moderately severe or severe at baseline in their SMMSE (*n* = 87). Cluster grouping (Severe, Moderate and Mild) was based on previously described method using baseline activities of daily living and psychiatric behaviour assessment scores only. SMMSE Standardised Mini-Mental State Examination.

The characteristics of each cluster are summarised in Table 4. The distribution of severity in the corresponding cognitive score (SMMSE) per cluster, is illustrated in Fig. 3. The severe cluster had the highest proportions of severe scoring SMMSE conversely, the mild cluster showed the highest proportions of normal and mild SMMSE scores.

**Table 4:** Summary of cluster demographics

Cluster	Diagnosis			Sex		Age group					House occupancy		Count (*n* = 87)
	%Alzheimer’s Disease	%Mild cognitive impairment	%Other	%Male	%Female	%50–59	%60–69	%70–79	%80–89	%90+	%Lives alone	%Lives with proxy-rater	
Severe	76.47	…	23.53	52.94	47.06	…	17.65	41.18	29.41	11.76	23.53	76.47	17.00
Moderate	92.37	3.85	3.85	57.69	42.31	…	3.85	15.38	50.00	30.77	42.31	57.69	26.00
Mild	54.55	13.64	31.82	56.82	43.18	2.27	4.55	43.18	38.64	11.36	31.82	63.64	44.00

Percentage of each characteristic and demographic that is present within each cluster group: Severe, Moderate and Mild.

**Fig. 4:**
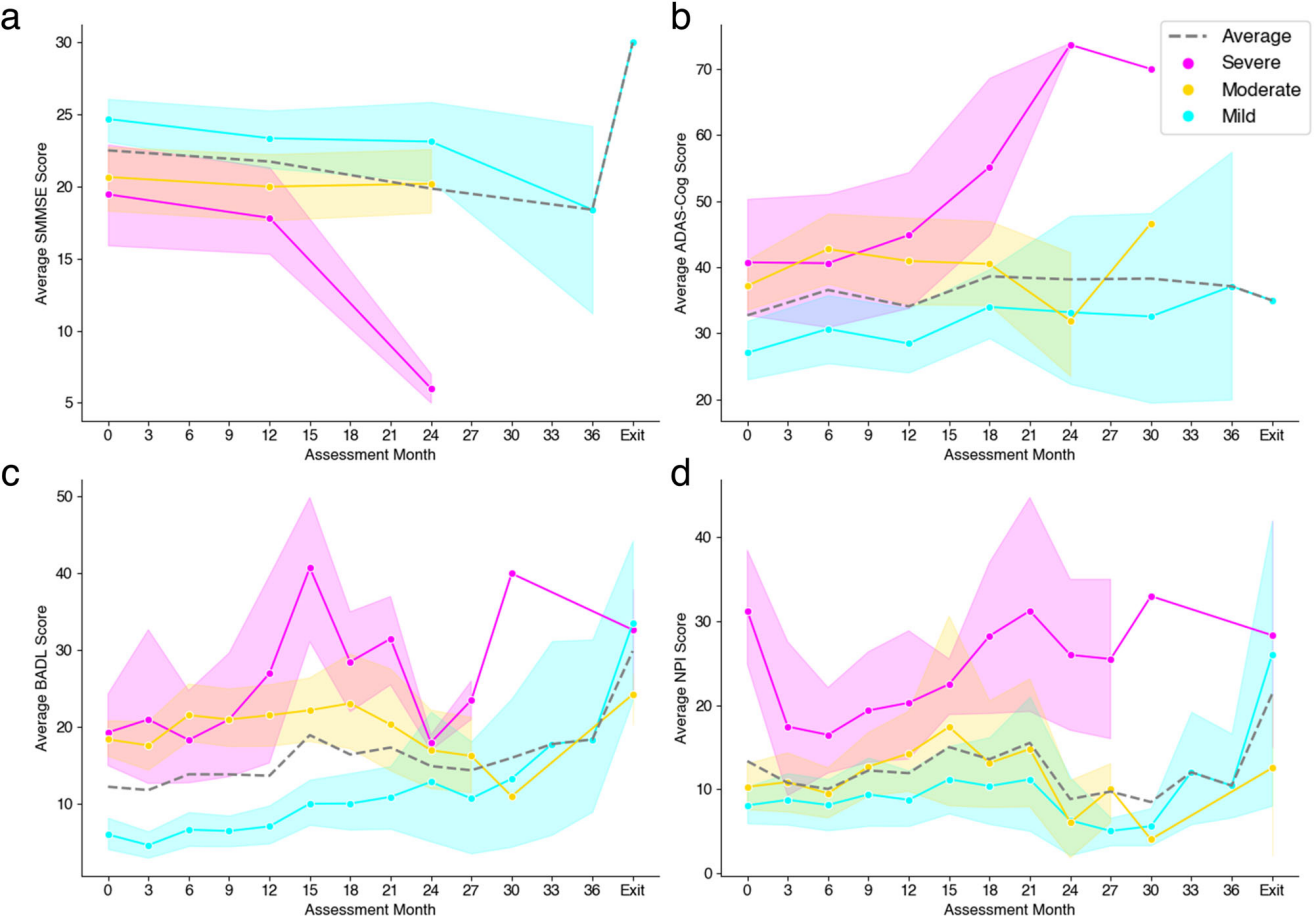
Average in-home assessment scores per cluster group across study time points. Average raw scores per assessment over the assessment time-points, including the exit timepoint, per cluster (*n* = 87). **a** Average cognitive SMMSE scores per cluster. **b** Average cognitive ADAS-Cog scores per cluster. **c** Average activities of daily living assessment scores per cluster. **d** Average psychiatric behaviour assessment scores per cluster. The average score per scale for the whole cohort is indicated by the grey dashed line in each subplot. Each of the solid lines represent a cluster group. NPI refers to the psychiatric behaviour assessment and BADL refers to the activities of daily living assessment. SMMSE Standardised Mini-Mental State Examination, ADAS-Cog Alzheimer’s Disease Assessment Scale Cognitive Subscale, BADL Bristol Activities of Daily Living, NPI Neuropsychiatric Inventory.

We compared these clusters according to their scores over time in each assessment scale to understand decline in the cohort (Fig. 4). Only a small sub-group of the cohort had completed at least two SMMSE scales throughout the study (*n* = 23) and there were 60 participants that completed at least two ADAS-Cog scales. The clustering only used the baseline scores although, distinct severity patterns can be identified, that continue throughout the study. Towards the end of the study, it is important to note the severe and moderate clusters became smaller in number, as seen by reduction in variation whereas, the mild cluster remained well populated. This was due to withdrawal of participants from the study.

The mild and severe clusters remained separate from one another throughout the study, for cognitive scales, behavioural symptoms and difficulties in activities of daily living. In particular, the severe cluster was the group that evidenced the greatest overall decline. The moderate and mild clusters showed a similar overlap for SMMSE cognitive assessment (Fig. 4a) and psychiatric behaviour assessment (Fig. 4d) throughout the study. For ADAS-Cog (Fig. 4b) and activities of daily living assessments (Fig. 4c), the moderate and severe cluster were of a similar profile at baseline however, as the study continued, the severe cluster evidenced a trajectory of decline.

Measuring cognition, behavioural symptoms and activities of daily living over time are pertinent to identifying decline of the cohort. We employed a mixed effects linear regression analysis to investigate this. Details of these results can be seen in Supplementary Fig. 6. Fixed effects for this model included the assessment time-points but, also whether participants had withdrawn, due to health-care-related progression including, care home admission, deterioration and death. Exit assessments for those withdrawn from the study were omitted from this analysis, due to risk of bias. Often, once participants had either passed away, gone to care homes or otherwise, proxy-raters who acted as carers, had a change in perspective and responses in exit assessments differed greatly from usual trajectory. In summary, results from this showed that there were considerable changes from baseline to all time points for SMMSE and ADAS-Cog (*p* < 0.01). Only activities of daily living assessments showed substantial changes from baseline after the 6-month time-point (*p* < 0.05). No significant changes were seen with psychiatric behaviour assessment or proxy-rater well-being assessment scores. Progression of proxy-rater well-being assessment scores over time could not be easily distinguished across the clusters and are illustrated in Supplementary Fig. 7.

### Profiling clusters—broader healthcare-related data

**Fig. 5:**
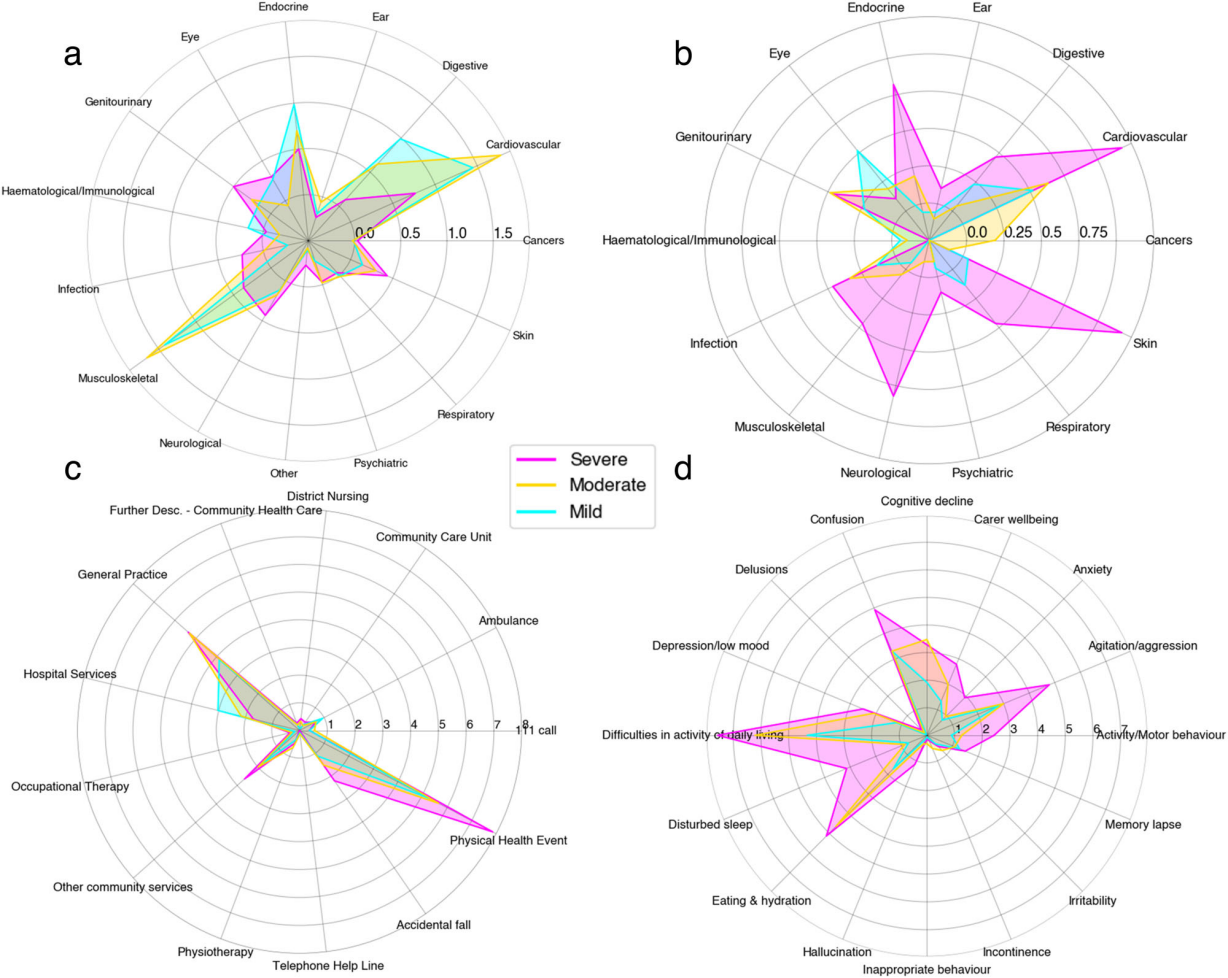
Average cumulative sum of healthcare-related data per cluster group. For each cluster group, Severe, Moderate and Mild, we calculated the average cumulative sum of: (**a**) comorbidities before baseline, (**b**) comorbidities after baseline, (**c**) healthcare events and encounters, and (**d**) behavioural observations, per cluster (*n* = 87). Distinct patterns of behaviour, comorbidities and healthcare events are illustrated for individual clusters.

Dementia progression can be influenced by existing comorbidities. Comorbidities were recorded directly from participants’ electronic health care records, for details on categorisation, see ‘Methods’ and also Supplementary Notes—Broader Healthcare-related Data. A total of 14 different comorbidity categories were observed with 753 individual comorbidity diagnoses. Figure 5 illustrates the average sum of (a) comorbidities diagnosed before baseline, (b) comorbidities diagnosed after baseline, (c) encounters with healthcare and (d) behavioural observations, per cluster. Information about participants’ general health and comorbidities is important in order to capture wider domains of dementia care.

On average, before the study began, the severe cluster had the highest number of comorbidities than any other cluster. Comorbidities that occurred consistently before and after baseline, were on average, more varied and higher in the severe cluster including, infections, neurological and skin/dermatological morbidities. The severe cluster also had the highest percentage (35%) of participants who had withdrawn due to healthcare-related reasons. Whereas, only 11% of the mild cluster withdrew and showed the highest incidence consistently, before and after diagnosis, in haematological/immunological morbidities.

On a regular basis, we measured participants’ interactions with healthcare services and how often they occurred, verifying this with electronic healthcare record data. On average, the severe cluster showed the highest incidence of physical health events such as infections, illnesses or medical ailments. The mild cluster showed highest incidence of hospital services and use of ambulance services. The severe and moderate cluster were equally, on average, interacting with general practitioners more so than the mild cluster. The severe cluster showed the highest incidence in all behaviours except inappropriate behaviour, incontinence, irritability and cognitive decline, all of which were on average seen more in the moderate cluster.

## Discussion

Tracking or monitoring the changes in everyday life and changes in behaviour in PLWD is not commonly implemented in clinical and care pathways in dementia. It is necessary to provide more enhanced care, to first understand the progression of PLWD in a natural home setting before implementing an intervention to improve symptoms and function. We have described how behaviour and difficulties of PLWD, progress over time. We have used the routinely collected in-home assessment data and applied unsupervised machine learning methods to identify patterns and sub-groups in the data. We clustered this cohort based on psychiatric behavioural symptoms and activities of daily living at baseline to identify groups of severity, that correspond to varying trajectories of decline. We contextualised these groups using self-reported interactions with healthcare services and changes in behaviour in conjunction with data from electronic healthcare records. Combining the cluster analysis and electronic healthcare records, we highlighted the frequent events and interactions with healthcare care services, in each sub-group. For new participants, we can assess their similarity of their baseline data to each subgroup. We can then use the subgroup profiles to identify and approximate the care requirements of the new participants. This work lays the foundation for incorporating regular monitoring of these behaviours and symptoms into routine, clinical dementia care to inform tailored interventions. The findings of this study currently provides insights to support clinicians and improve patient care of PLWD in an ongoing dementia health management cohort study.

In this cohort, the severity of participants’ dementia was a clear contributor to the evidenced behaviours and neuropsychiatric symptoms. In support of other work, we found that the most common symptoms for PLWD in the whole cohort were apathy and depression^3^. Over longer periods of time, for at least 6 months, changes in eating and sleeping behaviours were present in over half of the cohort (Fig. 1). Extensive studies have established problems with sleep and changes in sleep behaviours as a risk factor and highly prevalent in dementia^45,46^. However, the more severe participants in this work evidenced more difficult behaviours, including hallucinations and delusions, that were less common in those with ‘normal’ or ‘mild’ cognitive scores (Fig. 1). More difficult behaviours, such as these, require more intervention from families/carers and can lead to greater impact on healthcare services^47^. This not only infers cost to healthcare but also a greater psychological burden to those who care for the individual. Similarly, we found that proxy-raters of participants who experienced more difficult psychiatric behaviour had the most reported abnormal depression and anxiety. Consequently, understanding the carers’ interpretation of behavioural and psychological symptoms may alleviate costs associated with carer distress^48^. Several other longitudinal cohorts of PLWD including the ADAMS study^49^ and Bennett and colleagues^50^ examine the progression and prognosis amongst PLWD. However, the existing studies are often conducted in clinics and not within the home settings. Our work utilises in-home assessments, revealing a more novel and representative view of progression.

Targeting behavioural and psychological symptoms and difficulties in activities of daily living earlier in dementia care improves behavioural symptoms, including neuropsychiatric behaviours and reduces carer burden^51^. Clearly, behaviours and psychiatric symptoms are not the only changes that PLWD experiences. They also experience a huge detriment to their independence and as part of this, how they are able to live on their own and perform basic activities of daily living. In this cohort, the same pattern was seen where participants became more reliant on others for self-care and daily activities, including dressing themselves when they scored ‘severely’ in their cognitive assessment (Fig. 1). More ‘mild’ or ‘normal’ scoring cognitive severity showed difficulties with other activities, for example, shopping however, showed less difficulty with dressing or hygiene tasks. Exploring these discrepancies between cognitive abilities in a larger population, would allow us to develop flags where participants begin to transition from different levels of difficulty with living, acting as personalised indications, for more support and assistance from healthcare services. Most importantly, one must consider the burden these changes in ability and independence have on those who care for PLWD. This cohort showed that those with earlier stages of dementia (‘normal’ and ‘mild’ cognitive scores) showed a higher level of anxiety than those more cognitively impaired. Perhaps those who are more mild, have more insight and awareness into their situation and future and rely on their relatives and family members more. The initial changes in independence and the transition into diagnosis is a critical stage to identifying and managing anxiety of those caring for PLWD.

Critical to this translation to clinic, we are able to contextualise progression with regards to longitudinal healthcare data, including comorbidities and self-reported interactions with healthcare services. Arguably, comorbidities diagnosed after baseline Fig. 5b were more representative of a typical health profile of PLWD. In particular, the severe cluster was diagnosed with more comorbidities attributed to dementia risk including cardiovascular (this was inclusive of stroke) and comorbidities related to the ear (including hearing loss). This group were identified as having the most physical health events that were self-reported, that also corresponded to infection-related comorbidities from electronic healthcare records. This group also reported the highest number of falls and encounters, on average, with general practitioners, district nursing and other community services. Interestingly, this was not the group that encountered the most visits with ambulances, hospitals or 111 calls, inconsistent with other findings^52^. Although, this group did exhibit the highest number of and most varied behaviours reported by participants themselves or their proxy-raters. Perhaps this discrepancy was due to the lack of self-reporting from participants or their proxy-raters; however, in this context, hospital services can also include routine appointments and surgeries that were pre-scheduled and not emergencies only. Conversely, it is possible that more interactions with community-based services may mitigate the downstream effect on hospitals^53^. Individuals who are less cognitively impaired may not access community services as frequently. Conducting this analysis on a larger, population-level cohort would allow us to develop this further, as comorbidity management in PLWD is often highly complex^54^. Other home-based studies in the literature such as Dodge et al.^55^, explored the use of in-home sensors and cognitive assessments. Although these studies provided methodologies for technical developments for care solutions, they did not offer clinically applicable insights into wider effects of dementia progression^55^. On the other hand, studies that analysed healthcare service use in dementia patients were either limited to hospital or clinical settings only or lacked the longitudinal aspect, that is seen in this work^56,57^. Our study fills this gap by combining these data sources, allowing us to explore how daily functional changes, behaviours and interactions with the healthcare system influence long-term dementia care management.

It is likely that the reporting of behaviours in this cohort using activities of daily living and psychiatric behaviour assessment questionnaires results in more frequently observed symptoms and changes. Nonetheless, a fundamental hindrance to translating this to clinical care includes the reliance on resources and tools that would not be scaleable to a larger population for existing community health services. Despite this, we provide a longitudinal overview of how this cohort of PLWD differ from one another in multiple aspects of their dementia, in a non-interventional home setting.

Unsupervised clustering methods provided a preferable separation as opposed to simply ranking the participants based on their performance in one assessment scale or another. Specifically, we evaluated similarity based on the ratio of the sum of between-clusters and inter-cluster dispersions using the Calinski-Harabasz index. Other machine learning models have been used to predict dementia severity from self-reported patient symptoms, including behaviour and activities of daily living^58,59^. These also highlight the importance of everyday functioning and behaviours to PLWD and their families. Despite this, studies often lack a comparison or contextual embedding of wider healthcare data that we have focused on capturing here.

This study includes data from 141 participants. The clustering analysis contained 87 participants, all of whom had the measures that were used for the analysis. It is important to note that the focus of our investigation was not on achieving statistical power through sample size but rather on exploring the relationships and development of symptomatic changes for PLWD. Despite the smaller sample size, it includes a broad range of measures and multiple time point assessments, that provide a unique opportunity to gain insights into functional, behavioural and psychological symptoms in PLWD. Furthermore, the qualitative or exploratory nature of our analysis allowed for in-depth examination and rich data interpretation, compensating for the inherent limitations of sample size. This cohort did not include large socioeconomic diversity and contained data with repeated measures. Data collected from participant’s devices in the home often contained large amounts of missing data. However, this was not imputed or altered as we wanted to evaluate what could be identified from real-world data without alteration. This was an exploratory analysis to understand whether real-world data, without processing, could be linked to measured behavioural and psychological symptoms and difficulties in activities of daily living. Alternative scales, including the BEHAVE-AD scale, were specific to only AD and not other types of dementia; therefore, Neuropsychiatric Inventory was deemed more appropriate in this setting^60^. The ADAS-Cog is traditionally only used in research for patients with AD and no other diagnoses. Expanding the study’s scope to encompass diverse dementia types and refining methodologies will further enhance its clinical relevance and applicability.

A major advantage of the proposed solution is the ability to adapt to the current clinical pathways. The current dementia care is mainly focussed on responding to the acute needs of PLWD when events occur. This study offers a new approach and insights into early assessments and timely interventions. Existing studies have highlighted the importance of estimating care needs, to improve quality of life of PLWD and reduce the pressure on carers and healthcare services. The current practice largely relies on expert knowledge and heuristics to estimate these care needs. We present existing clinically validated assessments, collected routinely at home, can be used to transform care assessment and trajectory analysis of PLWD. We have utilised a large collection of longitudinal data to develop and validate the applicability of our approach in real-world settings.

To conclude, regular monitoring of functional ability and psychiatric behaviours in PLWD offers an opportunity to identify periods of change or decline, providing an interventional window otherwise not identified in current care settings. Our findings offer significant implications for improving healthcare support for PLWD. We present the importance of home-based functional monitoring alongside conventional healthcare service interactions. This will help to develop more effective, personalised care plans for dementia and estimate healthcare needs over time.

## Supplementary information

Supplementary Materials

Description of Additional Supplementary Files

Supplementary Data 1a

Supplementary Data 1b

Supplementary Data 1c

Supplementary Data 2

Supplementary Data 3

Supplementary Data 4

Supplementary Data 5a

Supplementary Data 5b

Supplementary Data 5c

Supplementary Data 5d

Reporting Summary

## Data Availability

Data for tables and figures are provided with the manuscript. Source data for Fig. 1 can be found in Supplementary Data 1a, b and c. Source data for Figs. 2 and 3 can be found in Supplementary Data Fig. 2 and Supplementary Data Fig. 3, respectively. Source data for Fig. 4 can be found in Supplementary Data Fig. 4. Source data for Fig. 5 can be found in Supplementary Data Fig. 5a, b, c and d. Further reasonable data requests can be made directly to the UK Dementia Research Institute, Care Research & Technology Centre at ukdri_crt@imperial.ac.uk.

## Abbreviations

ADAS-Cog: Alzheimer's Disease Assessment Scale—Cognitive Subscale
BADL: Bristol Activities of Daily Living Scale
HADS: Hospital Anxiety and Depression Scale
NHS: National Health Service
NPI: Neuropsychiatric Inventory
PLWD: People Living with Dementia
SMMSE: Standardised Mini-Mental State Examination

## Code availability

The code used in this study cannot be made publicly available due to proprietary restrictions between partners in the Minder Health Management Study. However, we have provided detailed methodological explanation to ensure reproducibility of the results. Please contact c.walsh22@imperial.ac.uk for any further enquiries.
