# Supplementary material for: Longitudinal study of care needs and behavioural changes in people living with dementia using in-home assessment data
Source: Commun Med (Lond). 2025 Jan 10;5:14. doi: 10.1038/s43856-024-00724-3 (PMC11724125; doi:10.1038/s43856-024-00724-3)
Supplement: Supplementary file 1 — Supplementary Materials [file 43856_2024_724_MOESM1_ESM.pdf]

Supplementary Notes

Relationships between Behavioural/Function and Physiology/Medication

We also collected data from devices in the homes of each participant (Supplementary Table 1) and set out to determine whether assessment scores were associated with medication dose or monthly physiological measures from in-home devices. Devices used in this study were largely based on previous work from this group [59, 60].

Physiology data from in-home monitoring devices were aggregated per month for each participant so that it aligned with the period assessed in the psychiatric behaviour assessments (Neuropsychiatric Inventory) (one month). The activities of daily living assessment (Bristol Activities of Daily Living) assesses changes in the previous two weeks therefore, one month was inclusive of both. No imputation was used in this analysis for the physiology data from devices in participants' homes, to understand whether real-world, unimputed data related to similarly, unimputed assessment data. This meant that the population available to use in this analysis was small (n=37) however, this was limited to only participants that were on either psychotropic, dementia or sleep medication, who also had available physiology data, one month either side of their assessment dates. Multiple assessments per individual were included here (76 activities of daily living assessments, 77 psychiatric behaviour assessments). This restricted the analysis heavily therefore, use of imputation techniques in future, would allow us to maximise this to the wider cohort.

Spearman's rank correlation analysis was conducted for scores of each question of activities of daily living and psychiatric behaviour assessments with medications and physiological measures. Only psychotropic, dementia and sleep medications were selected for this analysis. Medication data was calculated using the dose that each participant was on at the time of each assessment and this was multiplied against the frequency at which they take the medications per day. This limited sample was due to availability of complete, consecutive, timely data, relevant to the specific time surrounding each assessment (within one month). Any psychotropic and dementia medications used in this analysis only included those where at least 15% of the cohort were prescribed and doses of each medication referred to the daily dose on the day of the assessment. Physiological measurements from devices in participants' homes were aggregated to one month before the assessment time-point. Results of these correlations can be seen in Supplementary Figure 3.

Of clinical interest, for activities of daily living, the greater the lower-bound night-time respiration rate, the less difficulty participants had with most activities (Supplementary Figure 3a, $p<0.05$). In addition, the higher the average heart rate from the sleep mat, the more difficulties participants experienced with mobility (Supplementary Figure 3a, $p<0.05$). The earlier participants went to bed, the more difficulty participants had with dressing themselves and maintaining hygiene. Referring to earlier observations from the cohort, those that reported greatest difficulty with dressing and hygiene scored severely in the SMMSE (Figure 1a, $p<0.05$). Higher daily doses of Mirtazapine, an anti-depressant, correlated strongly with difficulties in maintaining hygiene, brushing teeth, using the bath/shower, orientation to space and communication (Supplementary Figure 3a, $p<0.05$). How participants communicated also had a linear relationship with the severity of the SMMSE therefore, more severe scoring participants showed greater difficulty with communicating (Figure 1a, $p<0.05$). An increased average temperature recorded from thermometers were associated with more difficulties in orientation to space (Supplementary Figure 3a, $p<0.05$). Additionally, those that had greater difficulty orientating their surroundings, scored more severely in the SMMSE (Figure 1a, $p<0.05$).

For neuropsychiatric behaviours and questions of the psychiatric behaviour assessment, the higher the night-time heart rate and lower-bound heart rate, the less the symptoms of elation, aberrant motor behaviours and anxiety were reported (Supplementary Figure 3b). Conversely, more time spent in bed and the later participants woke up, the more frequently they reported depression, anxiety, aberrant motor behaviour and changes in eating behaviours. Anxiety was seen to be the most commonly reported symptom in participants scoring normal SMMSE but, depression and eating behaviours were the most common for those scoring moderately severe (Figure 1b). Higher daily doses of Mirtazapine were associated with anxiety, apathy, disinhibition and aberrant motor behaviours (Supplementary Figure 3b). Higher daily doses of Memantine were also associate with lower pulse rates at night across the cohort.

Broader Healthcare-related Data

We explored our cohort and how their medical histories and diagnosed comorbidities related to their dementia progression. On a regular basis, each participant or proxy-rater, self-reported information about participants' general health and well-being, regarding changes in psychiatric behaviour and interactions with healthcare services.

Splitting this data in regards to the sex and diagnosis of the cohort, gave a more thorough understanding of how these comorbidites were prevelant within our cohort. Supplementary Figure 4 displays the average sum of each comorbidity

according to the category of (a) gender and (b) dementia diagnosis.

To understand the relevance of these comorbidites in relation to dementia diagnosis, we selected only the first instance of each morbidity for all participants, disregarding repeat occurrences. We then calculated the average number of years before and after each participants' dementia diagnosis, that they were diagnosed with each comorbidity, (Supplementary Figure 5).

Evaluation of Clustering

The metrics for each clustering method used and their relative performance is displayed in Supplementary Table 2. For this cohort, the Calinski-Harabasz metric was used to evaluate the optimal method as, this measured how well defined the clusters were. From these results, K-Means clustering produced the highest Calinski-Harabasz score for two, three and four cluster groups. The optimal cluster grouping was three groups as this struck a balance between optimal metric evaluation but also clinical interpretation and granularity of the data.

Mixed Effects Linear Regression Analysis Results

For the cognitive scales used in this regression analysis, we included 138 SMMSE and 233 ADAS-Cog observations, both with 85 distinct groups and successful conversion. Mean group size for SMMSE was 1.6 whereas, ADAS-Cog was 2.7. There were 428 activities of daily living assessment observations and 426 psychiatric behaviour assessment observations, with 87 distinct groups and successful conversion, with a mean group size of 4.9.

Supplementary Tables

Supplementary Table 1: Summary of physiological measurements and devices used for in-home monitoring for People Living with Dementia (PLWD) and the corresponding frequency of each measurement. Each participant was given an electronic tablet to view their data, to which the devices were connected to. This work only focused on the sleep mat, weighing scales, thermometer and blood pressure cuff. (RR: Respiration Rate, HR: Heart Rate).

**Digital Marker**	**Monitoring Device**	**Frequency**
Blood Pressure	Blood Pressure Monitor	Once per day
Temperature	Thermometer	Once per day
Weight	Digital Weighing Scales	Once per day/week
Sleep RR and HR	Sleep Mat	Once per minute
Sleep Activity Monitoring<br>(time in bed, time sleeping, entry/exit)	Sleep Mat	Once per minute
Activity	Passive Infrared Sensors	Triggered by movement
Door Activity	Passive Infrared Sensors	Triggered by movement
Home Appliance Usage	Smart Plugs	Triggered by usage

Supplementary Table 2: Results of performance metrics for unsupervised clustering methods for baseline activities of daily living and psychiatric behaviour scores from the cohort. A total of 35 features were used, made of questions from each of the baseline activities of daily living and psychiatric behaviour assessments. Calinski-Harabasz was used as the performance metric to establish the best separated clusters. Experiment 1 was selected for clustering, highlighted in bold.

Experiment	Model	Hyperparameters	Evaluation Metric				No. of Clusters
			Calinski Harabasz	Davies Bouldin	Dunn Index	Silhouette Score	
**1**	**K-Means Clustering**	**Max iterations = 100<br>No. initiations = 10**	**18.52**	**2.15**	**0.66**	**0.19**	**3**
2	Gaussian Mixture Model	Max iterations = 100<br>Covariance type = full	16.17	2.12	0.69	0.14	3
3	Bayesian Gaussian Mixture Model	Max iterations = 100<br>Covariance type = full	16.08	2.21	0.69	0.15	3
4	Agglomerative Clustering	Linkage = ward	15.19	2.27	0.48	0.08	3

Supplementary Table 3: Mean and standard deviations for each feature in the clustering model. NPI refers to the psychiatric behaviour assessment and BADL refers to the activities of daily living assessment. (BADL: Bristol Activities of Daily Living Scale, NPI: Neuropsychiatric Inventory).

Feature	Cluster					
	Severe n = 17		Moderate n = 26		Mild n = 44	
	Mean	Standard Deviation	Mean	Standard Deviation	Mean	Standard Deviation
BADL Preparing Food	1.53	1.18	1.77	1.14	0.45	0.82
BADL Eating	0.29	0.47	0.04	0.20	0.02	0.15
BADL Preparing Drinks	0.71	1.10	1.19	1.06	0.27	0.69
BADL Drinking	0.12	0.33	0.08	0.27	0.00	0.00
BADL Dressing	0.82	0.95	0.73	0.92	0.07	0.25
BADL Hygiene	0.47	0.94	0.92	1.09	0.05	0.21
BADL Cleaning Teeth	0.59	0.94	0.42	0.70	0.00	0.00
BADL Bath/Shower	0.88	1.22	1.31	1.12	0.07	0.25
BADL Toilet	0.47	0.72	0.12	0.43	0.00	0.00
BADL Transfers	0.47	0.62	0.08	0.27	0.02	0.15
BADL Mobility	1.00	0.94	0.81	0.90	0.43	0.79
BADL Orientation to Time	1.24	0.56	1.12	0.71	0.45	0.76
BADL Orientation to Space	1.29	0.69	0.88	0.52	0.36	0.53
BADL Communication	0.82	0.88	0.31	0.68	0.20	0.55
BADL Telephone	1.06	1.09	1.15	0.88	0.07	0.25
BADL Housework/Gardening	1.29	1.16	2.00	1.20	0.34	0.64
BADL Shopping	1.53	1.18	1.62	0.94	0.36	0.69
BADL Finances	1.12	1.17	1.15	0.92	0.09	0.29
BADL Games/Hobbies	1.82	1.01	1.23	0.91	0.23	0.57
BADL Transport	1.76	1.15	1.65	0.94	0.43	0.76
BADL Total Score	19.29	8.98	18.58	6.44	3.93	3.48
NPI Delusions	2.24	2.28	0.35	1.57	0.09	0.47
NPI Hallucinations	1.65	2.32	0.00	0.00	0.11	0.49
NPI Agitation/Aggression	1.71	1.90	0.23	0.65	0.59	1.21
NPI Depression/Dysphoria	2.59	3.32	1.35	1.70	1.00	1.99
NPI Anxiety	3.00	2.35	1.04	1.15	0.43	1.04
NPI Elation/Euphoria	0.88	1.65	0.15	0.61	0.07	0.45
NPI Apathy/Indifference	4.06	3.25	3.27	3.29	1.09	2.33
NPI Disinhibition	1.41	2.03	0.81	1.33	0.36	1.14
NPI Irritability/Lability	2.53	3.37	0.27	0.53	0.98	1.93
NPI Motor Disturbance	3.12	2.67	0.12	0.33	0.73	2.19
NPI Sleep	2.12	2.67	1.00	2.06	1.32	2.55
NPI Appetite/Eating	4.12	4.09	2.27	3.13	1.52	3.36
NPI Total Score	29.41	13.68	10.85	6.45	8.30	8.37
NPI Total Distress Score	18.35	8.65	7.42	3.96	4.80	4.48

Supplementary Table 4: Summary of Dunn's post-hoc test for pairwise comparisons of each cluster, for each feature used in the K-Means clustering model. All values indicated in bold are significant (* = p<0.05, ** = p<0.01, *** = p<0.001). NPI refers to the psychiatric behaviour assessment and BADL refers to the activities of daily living assessment. (BADL: Bristol Activities of Daily Living Scale, NPI: Neuropsychiatric Inventory).

Feature	Severe vs Mild	Severe vs Moderate	Mild vs Moderate
BADL Preparing Food	**1.35E-03***	5.39E-01	**7.62E-06*****
BADL Eating	**5.13E-04****	**2.74E-03***	8.16E-01
BADL Preparing Drinks	1.30E-01	**4.42E-02***	**1.81E-05*****
BADL Drinking	5.05E-02	5.35E-01	1.40E-01
BADL Dressing	**2.65E-04****	6.55E-01	**2.65E-04****
BADL Hygiene	8.68E-02	7.11E-02	**2.11E-05*****
BADL Cleaning Teeth	**7.03E-04***	6.33E-01	**9.36E-04****
BADL Bath/Shower	**5.47E-03***	1.13E-01	**1.94E-07*****
BADL Toilet	**2.32E-05*****	**2.55E-03***	2.80E-01
BADL Transfers	**1.97E-05*****	**7.43E-04***	5.00E-01
BADL Mobility	1.86E-02	5.02E-01	6.15E-02
BADL Orientation to Time	**2.02E-04****	5.74E-01	**3.41E-04****
BADL Orientation to Space	**1.85E-06*****	1.18E-01	**4.07E-04****
BADL Communication	**1.18E-03***	**1.17E-02***	5.72E-01
BADL Telephone	**3.64E-05*****	5.33E-01	**2.79E-08*****
BADL Housework/Gardening	**2.47E-03****	1.14E-01	**4.12E-08*****
BADL Shopping	**1.80E-04****	6.46E-01	**9.42E-07*****
BADL Finances	**1.10E-04****	5.30E-01	**1.46E-07*****
BADL Games/Hobbies	**3.87E-08*****	1.37E-01	**7.77E-06*****
BADL Transport	**2.95E-05*****	8.23E-01	**5.63E-06*****
BADL Total Score	**4.91E-09*****	8.97E-01	**4.61E-12*****
NPI Delusions	**2.28E-08*****	**1.45E-06*****	7.06E-01
NPI Hallucinations	**1.42E-05*****	**4.40E-06*****	4.36E-01
NPI Agitation/Aggression	**7.13E-03***	**7.86E-04***	2.60E-01
NPI Depression/Dysphoria	5.57E-02	4.12E-01	2.40E-01
NPI Anxiety	**1.92E-07*****	**4.97E-03****	**1.35E-02***
NPI Elation/Euphoria	**1.75E-04****	**3.90E-03****	4.89E-01
NPI Apathy/Indifference	**3.89E-05*****	3.55E-01	**3.39E-04****
NPI Disinhibition	**2.96E-03****	4.65E-01	**1.21E-02***
NPI Irritability/Lability	**2.60E-02***	**1.45E-03****	1.49E-01
NPI Motor Disturbance	**2.74E-06*****	**3.23E-06*****	6.48E-01
NPI Sleep	1.72E-01	1.18E-01	6.95E-01
NPI Appetite/Eating	**3.47E-03****	2.47E-01	**5.55E-02***
NPI Total Score	**5.43E-09*****	**7.27E-05*****	8.33E-02
NPI Total Distress Score	**1.40E-09*****	**1.64E-04*****	**2.52E-02***

Supplementary Figures

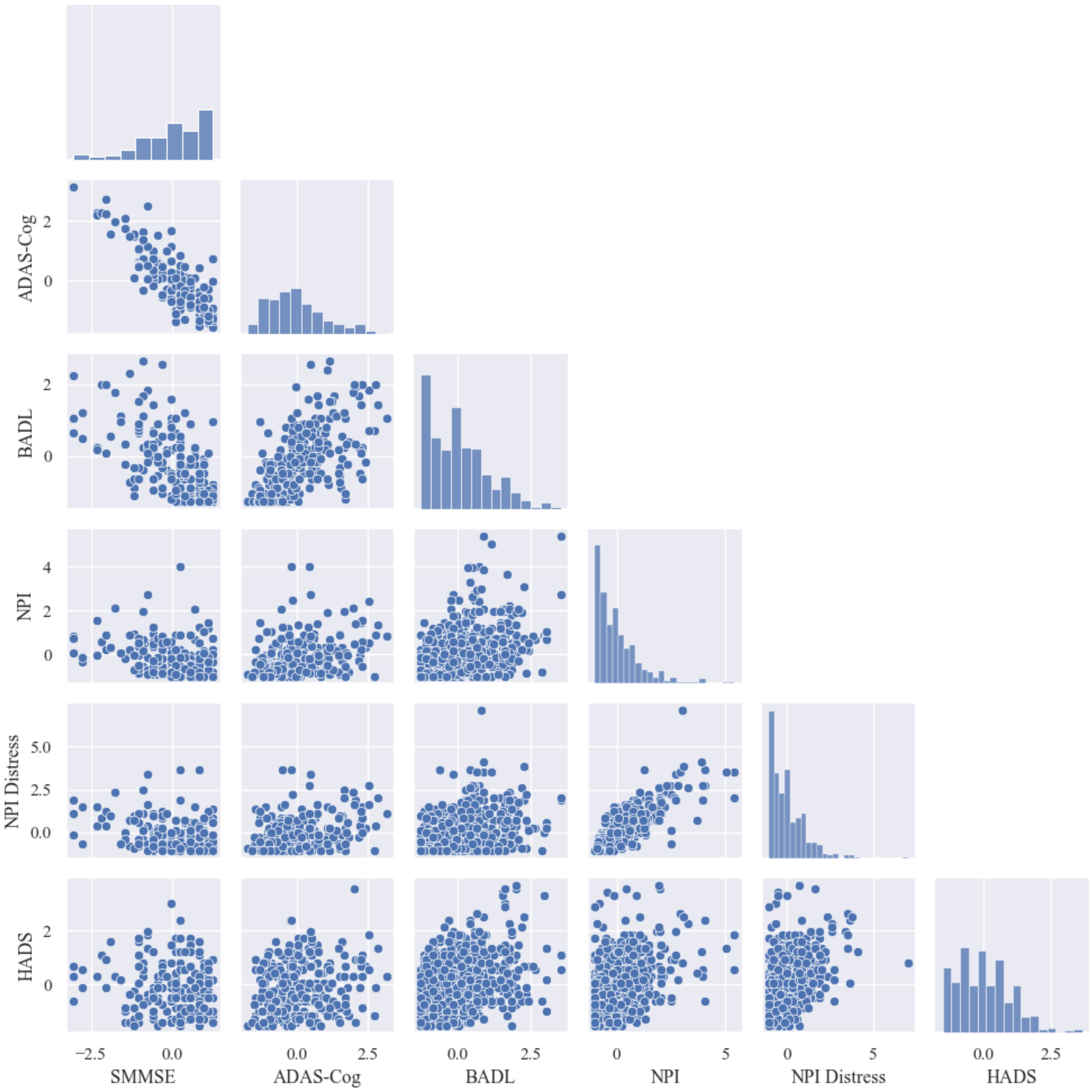

Supplementary Figure 1: Distribution of each assessment and their relationship with one another, for all assessments conducted throughout the study. Each plot contains one assessment shown as an individual point. Cognitive assessments include SMMSE conducted every twelve months and the ADAS-Cog every six months. NPI refers to the psychiatric behaviour assessment, NPI distress is proxy-rater distress associated with participants' psychiatric behaviour, BADL refers to the activities of daily living assessment and HADS refers to well-being of proxy-rater. (SMMSE: Standardised Mini-Mental State Examination, ADAS-Cog: Alzheimer's Disease Assessment Scale Cognitive Subscale, BADL: Bristol Activities of Daily Living Scale, NPI: Neuropsychiatric Inventory, HADS: Hospital Anxiety and Depression Scale).

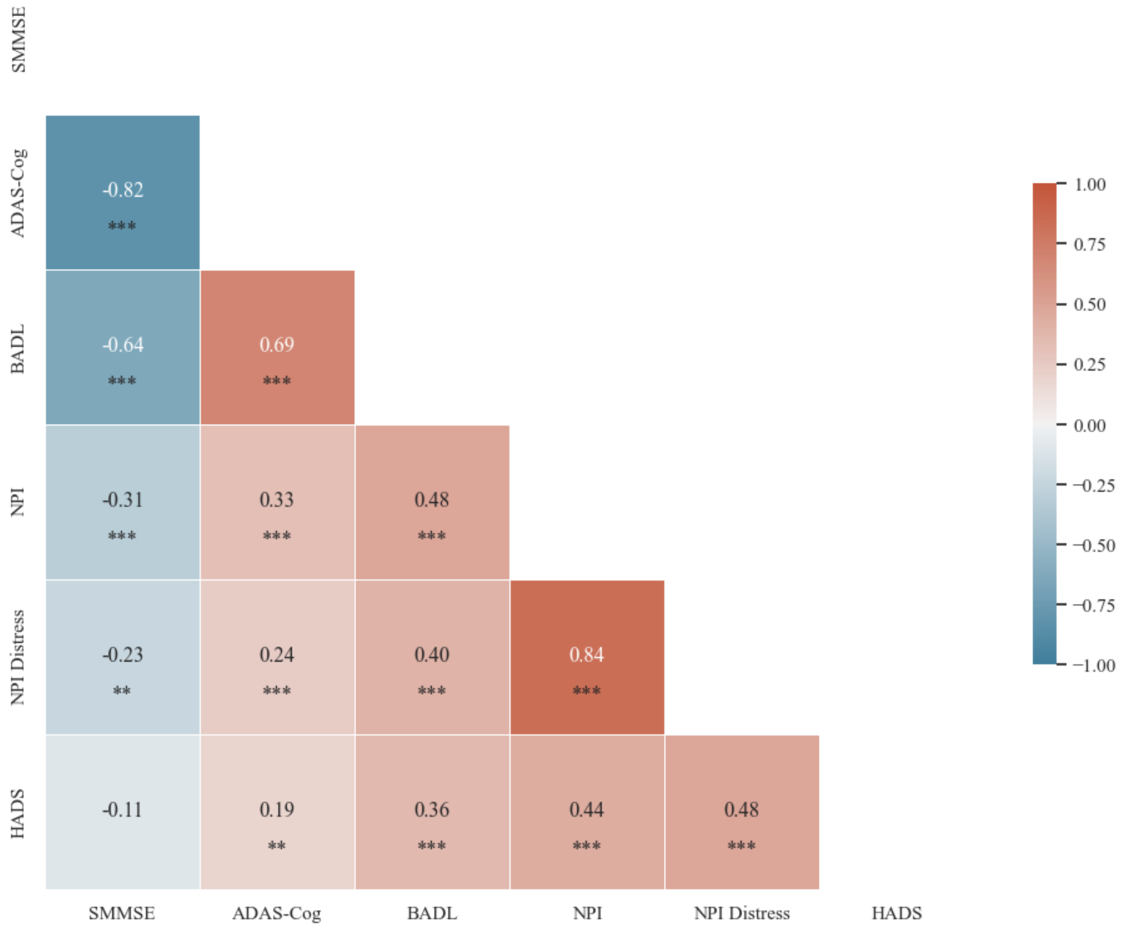

Supplementary Figure 2: Spearman's Rank Correlation analysis for all assessments conducted at baseline. Colour indicates the strength of the relationship therefore, red corresponds to a stronger positive correlation whereas blue refers to a stronger negative correlation. NPI refers to the psychiatric behaviour assessment, NPI distress is proxy-rater distress associated with participants' psychiatric behaviour, BADL refers to the activities of daily living assessment and HADS refers to well-being of proxy-rater. (SMMSE: Standardised Mini-Mental State Examination, ADAS-Cog: Alzheimer's Disease Assessment Scale Cognitive Subscale, BADL: Bristol Activities of Daily Living Scale, NPI: Neuropsychiatric Inventory, HADS: Hospital Anxiety and Depression Scale).

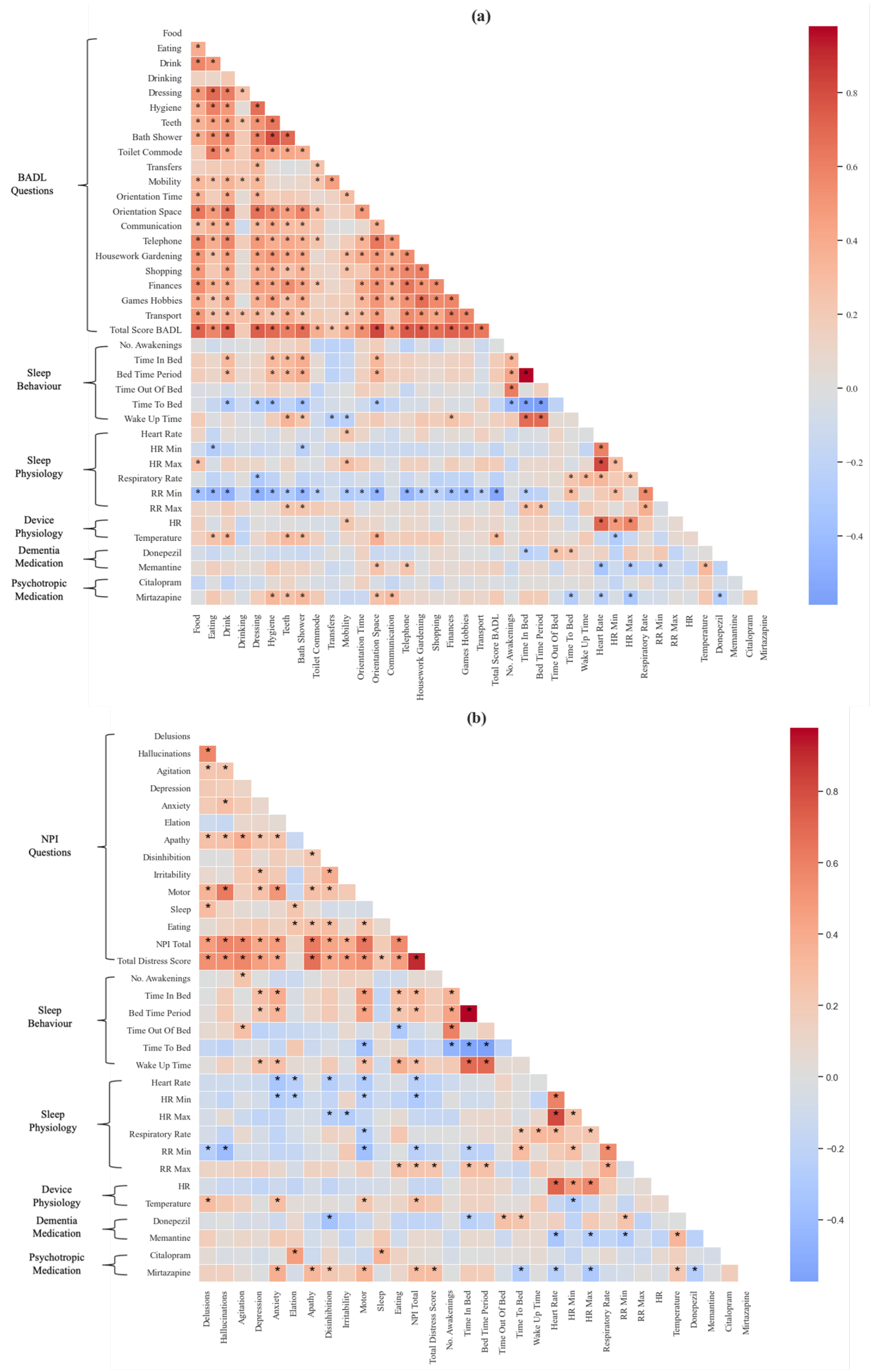

Supplementary Figure 3: Correlation analysis of activities of daily living and psychiatric behaviour with physiology data and medication doses. (a) BADL (n=76 time-points) scores and (b) NPI (n=77 time-points) scores and their relationship to measured sleep behaviour and physiology from sleep mat and blood pressure devices, as well as daily medication doses of dementia and psychotropic medications. Each square in the heat map indicates the strength of the correlation, the colour bar indicates the scale of the Spearman's correlation coefficient. Significant relationships below a 0.05 threshold are indicated by an asterisk. NPI refers to the psychiatric behaviour assessment and BADL refers to the activities of daily living assessment. (BADL, Bristol Activities of Daily Living Scale; NPI, Neuropsychiatric Inventory).

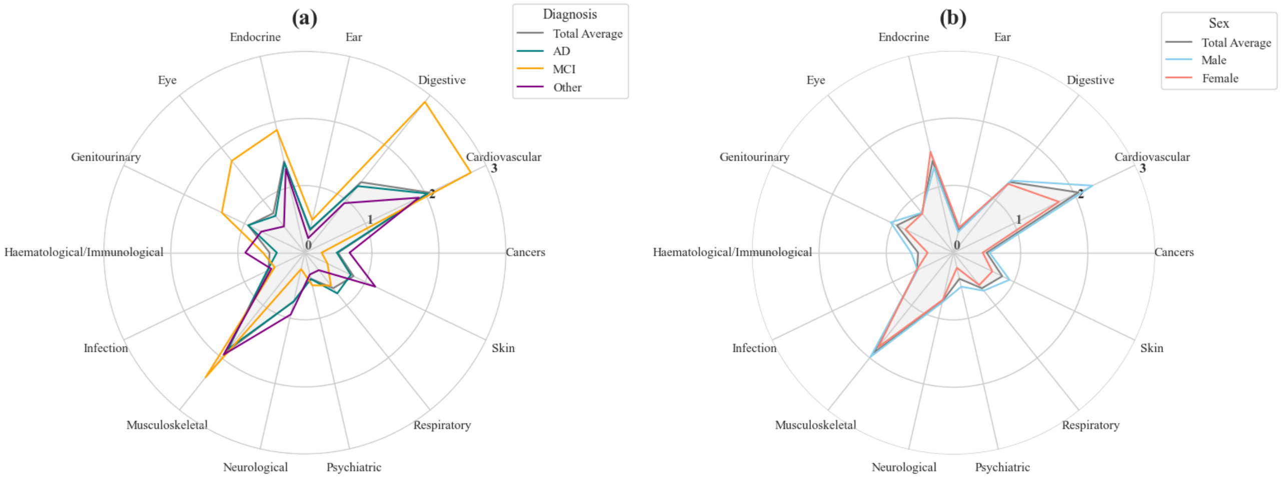

Supplementary Figure 4: Average sum of instance of comorbidity per category. (a) Average sum of comorbidity instances, grouped by dementia diagnosis. (b) Average sum of comorbidity instances grouped by sex, males and females in blue and pink, respectively (n=93). (AD: Alzheimer's Disease, MCI: Mild Cognitive Impairment).

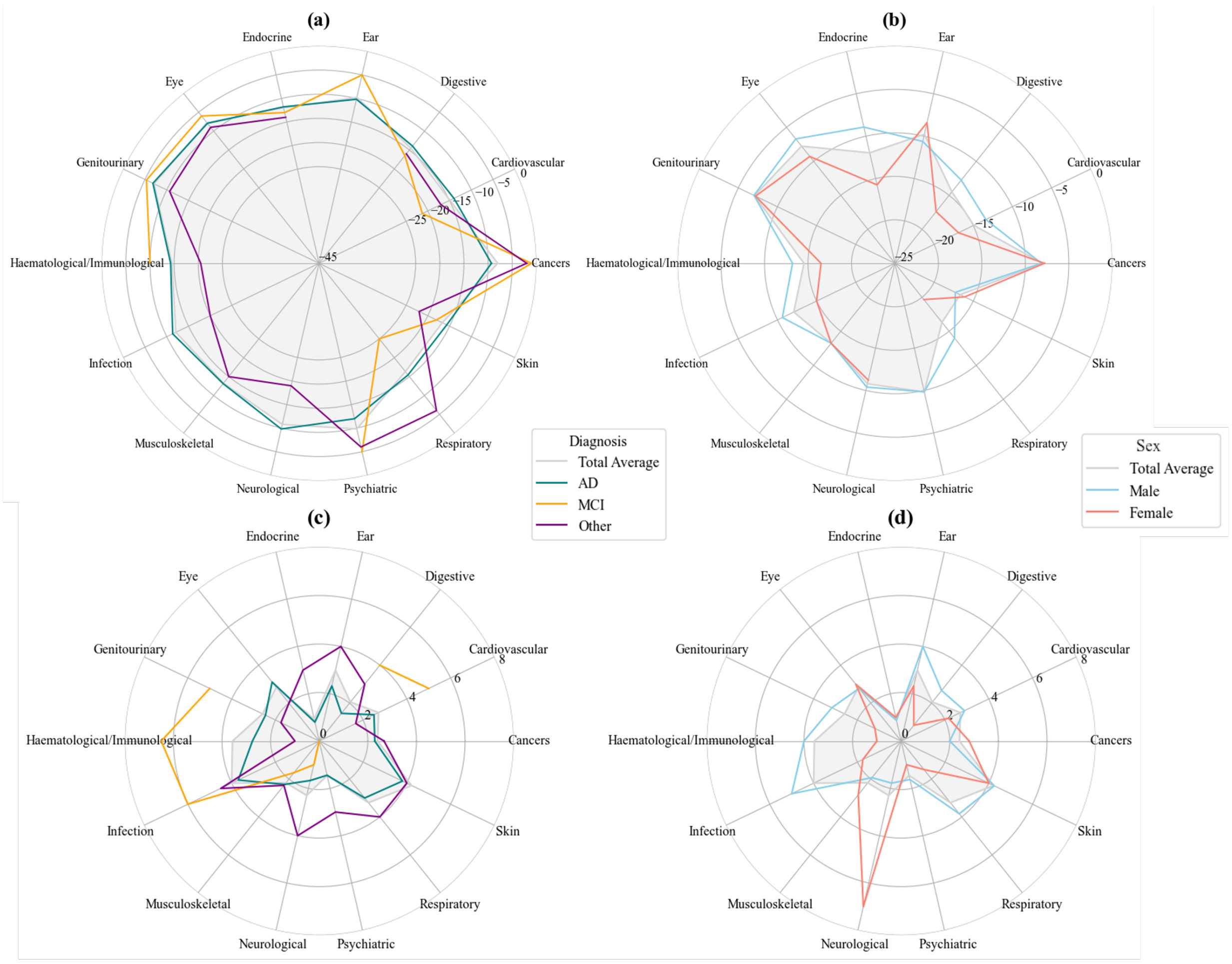

Supplementary Figure 5: Average number of years from diagnosis, for the first instance of each comorbidity. (a) Average years before, grouped by dementia type; AD, MCI and Other, in green, yellow and purple, respectively. (b) Each comorbidity and the average number of years they occurred before dementia diagnosis grouped by sex, males and females in blue and pink, respectively. (c) Average years after, grouped by dementia type. (d) Each comorbidity and the average number of years they occurred after dementia diagnosis grouped by sex. (AD; Alzheimer's Disease, MCI; Mild Cognitive Impairment).

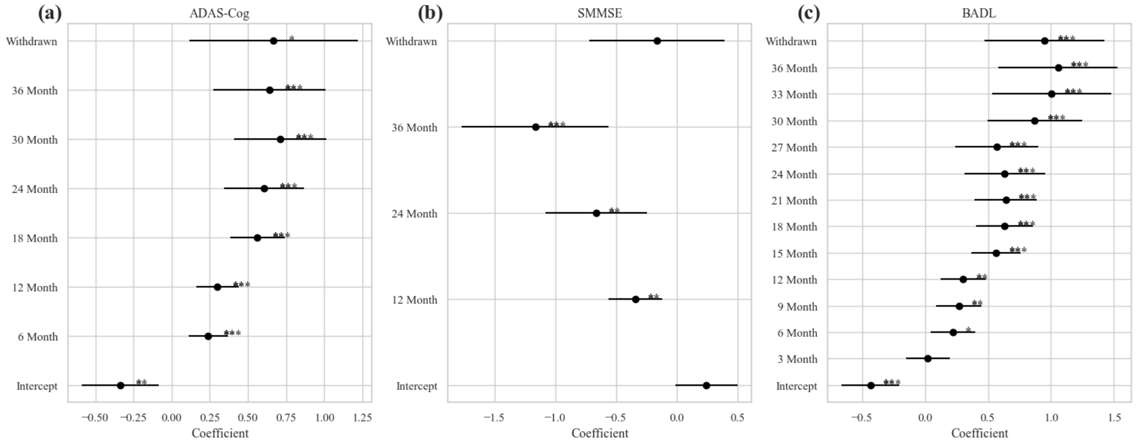

Supplementary Figure 6: Forest plots of coefficients from results of Mixed Effects Linear Regression Model for (a) ADAS-Cog, (b) SMMSE and (c) BADL. Significance annotations indicated by asterisks (* = p<0.05, ** = p<0.01, *** = p<0.001). ADAS-Cog and SMMSE refer to the cognitive assessments and BADL refers to the activities of daily living assessment. (ADAS-Cog: Alzheimer's Disease Assessment Scale Cognitive Subscale, BADL: Bristol Activities of Daily Living Scale, SMMSE: Standardised Mini-Mental State Examination).

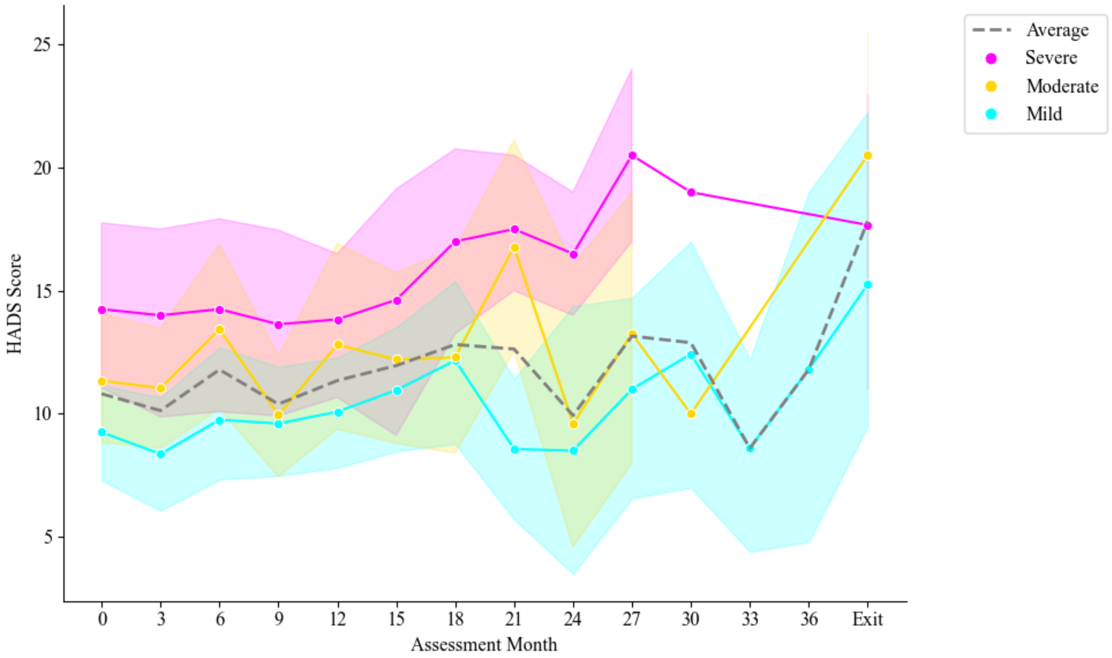

Supplementary Figure 7: HADS scores per cluster over the study time-points. Average for the whole cohort is indicated by the grey dashed line. HADS refers to well-being of proxy-rater. (HADS: Hospital Anxiety and Depression Scale).
