# Supplementary material for: Longitudinal study of care needs and behavioural changes in people living with dementia using in-home assessment data
Source: Commun Med (Lond). 2025 Jan 10;5:14. doi: 10.1038/s43856-024-00724-3 (PMC11724125; doi:10.1038/s43856-024-00724-3)
Supplement: Supplementary file 2 — Description of Additional Supplementary Files [file 43856_2024_724_MOESM2_ESM.pdf]

Description of Additional Supplementary Files

File name: Supplementary Data 1a, 1b and 1c.

File description: Source data for Figure 1

File name: Supplementary Data 2

File description: Source data for Figure 2

File name: Supplementary Data 3

File description: Source data for Figure 3

File name: Supplementary Data 4

File description: Source data for Figure 4

File name: Supplementary Data 5a, 5b, 5c and 5d

File description: Source data for Figure 5
