# Supplementary material for: Longitudinal study of care needs and behavioural changes in people living with dementia using in-home assessment data
Source: Commun Med (Lond). 2025 Jan 10;5:14. doi: 10.1038/s43856-024-00724-3 (PMC11724125; doi:10.1038/s43856-024-00724-3)
Supplement: Supplementary file 13 — Reporting Summary [file 43856_2024_724_MOESM13_ESM.pdf]

nature portfolio

Corresponding author(s): Ramin Nilforooshan, Chloe Walsh & Payam Barnaghi

Last updated by author(s): Oct 25, 2024

Reporting Summary

Nature Portfolio wishes to improve the reproducibility of the work that we publish. This form provides structure for consistency and transparency in reporting. For further information on Nature Portfolio policies, see our Editorial Policies and the Editorial Policy Checklist.

Statistics

For all statistical analyses, confirm that the following items are present in the figure legend, table legend, main text, or Methods section.

n/a	Confirmed	
☐	☒	The exact sample size (n) for each experimental group/condition, given as a discrete number and unit of measurement
☐	☒	A statement on whether measurements were taken from distinct samples or whether the same sample was measured repeatedly
☐	☒	The statistical test(s) used AND whether they are one- or two-sided<br>*Only common tests should be described solely by name; describe more complex techniques in the Methods section.*
☐	☒	A description of all covariates tested
☐	☒	A description of any assumptions or corrections, such as tests of normality and adjustment for multiple comparisons
☐	☒	A full description of the statistical parameters including central tendency (e.g. means) or other basic estimates (e.g. regression coefficient) AND variation (e.g. standard deviation) or associated estimates of uncertainty (e.g. confidence intervals)
☐	☒	For null hypothesis testing, the test statistic (e.g. F, t, r) with confidence intervals, effect sizes, degrees of freedom and P value noted<br>*Give P values as exact values whenever suitable.*
☒	☐	For Bayesian analysis, information on the choice of priors and Markov chain Monte Carlo settings
☐	☒	For hierarchical and complex designs, identification of the appropriate level for tests and full reporting of outcomes
☐	☒	Estimates of effect sizes (e.g. Cohen's d, Pearson's r), indicating how they were calculated

Our web collection on statistics for biologists contains articles on many of the points above.

Software and code

Policy information about availability of computer code

Data collection	The Minder dataset is obtained from the Minder Health Management Study (IRAS: 257561), an ongoing study collecting in-home data from people with an established diagnosis of dementia or other long-term frailty condition, at the UK Dementia Research Institute (UK DRI) Care Research & Technology Centre.
Data analysis	All analysis used in this work was done using Python (V3.9.13) and libraries used included Scikit-Learn version: 1.1.3, Pandas version: 1.5.1, NumPy version: 1.23.4 and visualisation was done using Seaborn version: 0.11.2.

For manuscripts utilizing custom algorithms or software that are central to the research but not yet described in published literature, software must be made available to editors and reviewers. We strongly encourage code deposition in a community repository (e.g. GitHub). See the Nature Portfolio guidelines for submitting code & software for further information.

Data

Policy information about availability of data

All manuscripts must include a data availability statement. This statement should provide the following information, where applicable:

- Accession codes, unique identifiers, or web links for publicly available datasets
- A description of any restrictions on data availability
- For clinical datasets or third party data, please ensure that the statement adheres to our policy

The data that support the findings of this study are available from the corresponding author on reasonable request. The code used in this study will be made available by the corresponding author on reasonable request.

Research involving human participants, their data, or biological material

Policy information about studies with human participants or human data. See also policy information about sex, gender (identity/presentation), and sexual orientation and race, ethnicity and racism.

Reporting on sex and gender	We do not collect information regarding participants' gender. However, we have reported on sex within the analysis and the larger cohort. Additional details of this can be found in supplementary material.
Reporting on race, ethnicity, or other socially relevant groupings	We do not report race or ethnicity, these were not part of the analysis.
Population characteristics	We have reported all of the relevant population characteristics.
Recruitment	People with multi-morbidity and or long term conditions were recruited through health and social care partners within the primary care network (GP practices) and community NHS trusts, urgent and acute care services within acute NHS trusts as well as social services. In addition, people with a diagnosis of dementia will be recruited from NHS community mental health teams for older adults and through specialist memory services at Surrey and Borders Partnership NHS Foundation Trust and within North-West London.<br><br>Inclusion criteria were as follows:<br>Frail adults and/or people living with neurological conditions/neurodegenerative disease.<br>Male or female 50 years of age and older at baseline.<br>Have sufficient functional English to allow completion of the assessment instruments.<br>Willing and able to provide informed consent.<br>Participants lacking capacity for informed consent were required to have a partner or caregiver who had known them for at least 6 months and was able to attend research assessments with them.<br><br>Exclusion criteria were as follows:<br>People with unstable mental state including severe depression, severe psychosis, agitation and anxiety at screening and baseline.<br>People with severe sensory impairment at screening and baseline.<br>Currently have active suicidal ideas.<br>People who are receiving treatment for terminal illness at screening and baseline.
Ethics oversight	The Minder study received ethical approval from the London-Surrey Borders Research Ethics Committee; TIHM 1.5 REC: 19/LO/0102; IRAS: 257561; ISRCTN71000991.46 All participants provided written informed consent.

Note that full information on the approval of the study protocol must also be provided in the manuscript.

Field-specific reporting

Please select the one below that is the best fit for your research. If you are not sure, read the appropriate sections before making your selection.

☐ Life sciences ☒ Behavioural & social sciences ☐ Ecological, evolutionary & environmental sciences

For a reference copy of the document with all sections, see nature.com/documents/nr-reporting-summary-flat.pdf

Behavioural & social sciences study design

All studies must disclose on these points even when the disclosure is negative.

Study description	The Minder study aims to support people living with dementia and other long-term health conditions, to live independently and avoid hospital admissions. This longitudinal study aims to observe trends in health-care-related events and hospitalisations, explore the impact of Minder Health Management on patients' and carers' experience of care. The Minder study aims to use the data to assess disease progression and correlate with real-world outcomes, cognitive function tests and other dementia markers.

Research sample	Frail adults and/or people living with neurological conditions/neurodegenerative disease<br>Male or female 50 years of age and older at baseline<br>Have sufficient functional English to allow completion of the assessment instruments<br>Willing and able to provide informed consent<br>Participants lacking in capacity must have a personal or professional consultee representative
Sampling strategy	A total of 677 activities of daily living assessments, 632 psychiatric behaviour assessments and 646 proxy-rater well-being assessments were available in the cohort. 109 participants had completed 296 ADAS-Cog scales and 118 participants had completed 194 SMMSE scales. 87 baseline activities of daily living (BADL), behaviour (NPI) and proxy-rater well-being (HADS) assessments were completed.
Data collection	Participants completed a dementia proforma at baseline, that consisted of information about demographics, including age and sex. Participants and proxy-raters completed multiple, regular assessment scales however, only five relevant assessment scales were selected for analysis. Fortnightly telephone calls were conducted to collect data about self-reported changes in behaviour and any interactions with healthcare services. We also used electronic healthcare record data to verify diagnoses and incidences of comorbidities for each participant.<br><br>Regular assessments were conducted by research assistants and included two standardised, validated cognitive assessments, the Alzheimer's Disease Assessment Scale Cognitive Sub-scale (ADAS-Cog) (14-item) every six months; and the Standardised Mini-Mental State Examination (SMMSE) every twelve months. Scaling of the cognitive assessments differs such that, the more severe the cognitive impairment, the higher the ADAS-Cog score; whereas, the lower the score for SMMSE, the more severely cognitively impaired the individual.<br><br>In addition, we used the previously validated Bristol Activities of Daily Living Scale as an assessment of activities of daily living and the Neuropsychiatric Inventory as a measure of psychiatric behavioural symptoms of \ac{PLWD}. \textcolor{blue}{From herein, we will refer to the Bristol Activities of Daily Living Scale (BADL) as the activities of daily living assessment and the Neuropsychiatric Inventory (NPI) as the psychiatric behaviour assessment}. The psychiatric behaviour assessment (Frequency*Severity) accounts for the neuropsychiatric symptoms that each participant experiences whereas, the psychiatric behaviour assessment distress score measures the proxy-rater distress according to how severe and frequent the symptoms of the participant are. Both the activities of daily living and psychiatric behaviour assessments were about the participant however, were completed by the proxy-raters.
Timing	Data used in this study is from the Minder Health Management Study (IRAS: 257561), spanning over three years (24/07/2020 to 01/09/2023). Participants were recruited on a rolling basis and therefore, this period covers all participants.
Data exclusions	We have excluded participants that did not have a proxy-rater. We excluded participants that did not have a diagnosis of dementia.
Non-participation	Of the 141 participants included in this study, 16 withdrew. Reasons included admission to care home, hospitalisation or deterioration in health.
Randomization	No randomization, this was an observational study only.

Reporting for specific materials, systems and methods

We require information from authors about some types of materials, experimental systems and methods used in many studies. Here, indicate whether each material, system or method listed is relevant to your study. If you are not sure if a list item applies to your research, read the appropriate section before selecting a response.

Materials & experimental systems

n/a	Involved in the study	
☒	☐	Antibodies
☒	☐	Eukaryotic cell lines
☒	☐	Palaeontology and archaeology
☒	☐	Animals and other organisms
☐	☒	Clinical data
☒	☐	Dual use research of concern
☒	☐	Plants

Methods

n/a	Involved in the study	
☒	☐	ChIP-seq
☒	☐	Flow cytometry
☒	☐	MRI-based neuroimaging

Clinical data

Policy information about clinical studies

All manuscripts should comply with the ICMJE guidelines for publication of clinical research and a completed CONSORT checklist must be included with all submissions.

Clinical trial registration	ISRCTN71000991
Study protocol	The Minder study protocol recieved ethical approval from the London-Surrey Borders Research Ethics Committee; TIHM 1.5 REC: 19/LO/0102; IRAS: 257561; ISRCTN71000991.46 . Protocol V 17.0 (21/08/2023).

Data collection

People with multi-morbidity and or long term conditions were recruited through health and social care partners within the primary care network (GP practices) and community NHS trusts, urgent and acute care services within acute NHS trusts as well as social services. In addition, people with a diagnosis of dementia will be recruited from NHS community mental health teams for older adults and through specialist memory services at Surrey and Borders Partnership NHS Foundation Trust and within North-West London.

Inclusion criteria for participation were frail adults and/or people living with neurological conditions/neurodegenerative diseases, including dementia, stroke and traumatic brain injury. Exclusion criteria included people with severe depression, severe psychosis, agitation and anxiety at screening and baseline. People with severe sensory impairment, active suicidal ideas and those receiving treatment for terminal illness at baseline were also excluded. For the purposes of this work, we only include participants with a diagnosis of dementia (n=141).

Participants were recruited from primary care, adult social care services and memory clinics across Surrey and Borders Partnership National Health Service (NHS) Foundation Trust and Hammersmith & Fulham Health and Care Partnership, North West London. This cohort included participants who had a confirmed diagnosis of dementia, a total of 141. Participants completed a dementia proforma at baseline, that consisted of information about demographics, including age and sex. Participants and proxy-raters completed multiple, regular assessment scales however, only five relevant assessment scales were selected for analysis. Fortnightly telephone calls were conducted to collect data about self-reported changes in behaviour and any interactions with healthcare services. We also used electronic healthcare record data to verify diagnoses and incidences of comorbidities for each participant.

Outcomes

This longitudinal study aims to observe trends in health-care-related events and hospitalisations, explore the impact of Minder Health Management on patients' and carers' experience of care. The Minder study aims to use the data to assess disease progression and correlate with real-world outcomes, cognitive function tests and other dementia markers.

Additional technical objectives were as follows:
Demonstrate a pilot showcase for prevention and early intervention by combining various data and by developing predictive models and analysis of the patients' data.
Demonstrate the development and implementation of technology enabled solutions and monitoring, control and alert services for people with long-term conditions.

Plants

Seed stocks

N/A

Novel plant genotypes

N/A

Authentication

N/A
